# Glass Fiber Reinforced Concrete as a Durable and Enhanced Material for Structural and Architectural Elements in Smart City—A Review

**DOI:** 10.3390/ma15082754

**Published:** 2022-04-08

**Authors:** Julia Blazy, Rafał Blazy, Łukasz Drobiec

**Affiliations:** 1 Department of Building Structures, Faculty of Civil Engineering, Silesian University of Technology, Akademicka 5, 44-100 Gliwice, Poland; lukasz.drobiec@polsl.pl; 2 Department of Spatial Planning, Urban and Rural Design, Faculty of Architecture, Cracow University of Technology, Podchorążych 1, 30-084 Kraków, Poland; rblazy@pk.edu.pl

**Keywords:** Smart City, glass fiber reinforced concrete, physical properties, mechanical properties, sustainable development, application, structural and architectural elements

## Abstract

The article highlights that glass fiber reinforced concretes (GFRC) can meet the requirements of Smart City better than ordinary concretes. The comprehensive discussion on GFRC composition is presented together with the review of glass fibers’ influence on various concrete properties. First of all, because of their bridging abilities, they can limit the width, length, and total area of cracks. Additionally, GFRC are characterized by enhanced tensile, flexural, and splitting strength; impact, abrasion, spalling, fire, and freeze-thaw resistance as well as ductility, toughness, and permeability. All of this positively influences the mechanical behavior, durability, and corrosion resistance of concrete elements. Moreover, decreased thermal conductivity allows for better energy performance from the building’s point of view. This results in cheaper structures both in manufacturing and maintaining even though GFRC are more expensive materials. However, mechanical properties enhance as long as sufficient workability and uniform fiber distribution are assured. From the environmental point of view, GFRC are eco-friendlier materials than ordinary concretes since their application can decrease the emission of CO_2_ by 17%. The article also describes the GFRC application fields and emphasizes the possibility of the creation of not only structural elements mainly intended for load transferring but also elements accompanying the building process, as well as elements of small architecture that make public spaces more attractive, durable, and safer. Owing to greater design and shaping freedom, GFRC can also better fulfill the needs of habitants of Smart City.

## 1. Introduction

A Smart City is a city that uses new technologies and solutions to improve the quality of ordinary life through public services. Currently, there is a tendency to divide smart cities into three generations which quite well reflects how the approach to cities and urban technologies has changed in the past few decades. The first-generation Smart City 1.0 focuses on new technologies and business makers who encourage cities to adapt their solutions to be more efficient in the management of urban organisms. It must be mentioned that very often city administration units are not prepared to use these advanced technologies. In Smart City 2.0, municipal authorities play a major role and they initiate changes and choose technologies and solutions to increase the inhabitants’ life quality. The most current generation Smart City 3.0, which has been functioning for a relatively short time, since approximately 2015, puts in the center the residents of the city. They co-create their cities, which means that implemented solutions should respond to the problems of inhabitants and be consulted with them. In this last model, more and more emphasis is placed on smart manufacturing, smart buildings, and smart environment. The smart idea should not only be implemented in the utility sphere, but also it should be present in the execution stage. Additionally, the idea of sustainable development is strictly connected with the suggestions of Smart City. One of the current most important global goals is to fulfill sustainability requirements in the concrete industry since it is responsible for 8–9% of total C0_2_ in the atmosphere [1]. Namely, during 1 kg of Portland cement production, 1 kg of C0_2_ is released into the atmosphere [1]. As a result, the possibility of manufacturing more durable building materials, slenderer concrete elements with extended service life are emphasized. According to this, glass fiber reinforced concretes (GFRC) can meet these needs better than ordinary concretes and as a result, have a lower environmental impact. Although, regarding GFRC which are a more costly materials than plain concretes (PC), it is essential to research both theirs reasonable structural use, as well as theirs physical and mechanical advantages.

**Figure 1:**
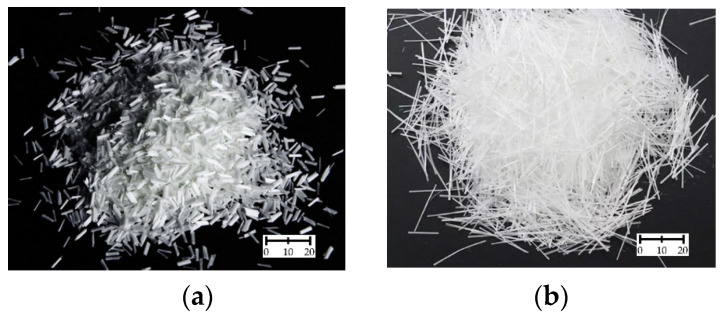
Glass fibers: (**a**) *l_f_* = 6 mm [10]; (**b**) *l_f_* = 24 mm [11].

Glass fibers (GF) began to be used around 1931 as a reinforcement of mortars and concretes [2]. They are obtained by pulling the molten glass mass through round holes, joining about 200–240 individual fibers into strands, and then by cutting them into smaller sections [3,4] (Figure 1). GF are characterized by their length (*l_f_*), diameter (*d_f_*), and slenderness (*l_f_/d_f_*). It must be mentioned that the fiber type and geometry have an influence on the mechanical and cracking resistance as well as durability and pore structure of concrete [5]. Depending on the chemical composition, properties, and application, different types of GF can be distinguished. The division with a short description is presented in Table 1. Because of the alkaline environment of a cement matrix, fibers are made of zirconium glass to have both alkaline and acid resistance. Thus, typically concrete contains fibers from E and AR-type glass. The volume weight of GF is about 2.5–2.7 g/cm^3^. They are characterized by high tensile strength (*f_ft_*), from 1200 to even 4800 MPa. Furthermore, GF have a much greater modulus of elasticity than synthetic fibers; however, smaller than steel and carbon fibers. They are resistant to high temperatures and begin to soften at approx. 700–900 °C [6]. The disadvantage of GF is their high sensitivity to water and poor alkali resistance in alkali environments [7]. This is because they wash away alkali metal salts and thus create fissures on their outer surfaces [6]. As a result, GF are protected against the negative effects of moisture in the environment in the process of silane preparation [8].

Regarding the production process of GFRC, there are three techniques to cast concrete elements reinforced with GF. The first possibility is called the hand spray-up method and this is how most of the ornamental precast elements and precast architectural cladding panels from GFRC are produced. Those mixes have usually higher fiber content (*V_f_*) between 4 and 6% and require careful expertise, special equipment, and experienced workers. Then vibration casting is carried out, which is a much simpler method, in which the GFRC premix is vibrated in the mold until it achieves consolidation. However, water-tight molds are required, and rock molds do not work well in this method. Finally, the sprayed premix method does not need so rigorous quality control as hand spray-up and strengths higher than those obtained in vibration casting can be achieved [9].

**Table 1:** Types of glass fibers [12,13].

Type	Description
E	Maximum alkali content 1% by weight, used when high strength and electrical resistivity are required, the basic material for reinforcement;
A	Low alkali resistance, used when the strength and electrical resistivity requirements are smaller than for E-glass;
C	High resistance to chemicals and corrosion;
D	Low dielectric constant, used in the electromagnetic industry;
R, S	Used when very high strength is required, for the production of textiles or composites for structural reinforcement, special applications;
AR	High strength and alkali resistance, used as concrete reinforcement.

This article introduces the solution for construction by the principles of the Smart City concept and sustainable development by using more advanced and smart materials. Moreover, it emphasizes the possibility of creating not only GFRC structural elements mainly intended for load transferring but also elements accompanying the building process, as well as elements of small architecture that make public spaces more attractive, durable, and safer. The work presented here is an interdisciplinary study since it involves the combination of civil engineering and architecture ideas. Regarding the research significance, no article considers the application of GF in these two scientific disciplines, which can interpenetrate each other.

## 2. Methodology

Studies analyzed in this review had to meet specific criteria and include relevant information. Keyword methodology according to which articles were selected is presented in Table 2. The eligible works were searched through databases like Scopus, Google Scholar, Research Gate, as well as MDPI journal search. First, titles and abstracts were taken into consideration, and afterward, the full paper was examined for suitability. The papers were excluded usually because they were written in a foreign language, included just brief information about properties and application of GFRC, considered only statical or numerical analyses, or referred to the glass fiber concrete polymers rather GFRC itself. Finally, from selected articles, the appropriate data were extracted and subjected to further analyses. From around 108 references including articles, conference papers, and books almost 85% were published between 2010 and 2022. Additionally, the search focused on companies dealing with the production of GFRC elements.

## 3. Description of GFRC Material Composition

In Table 3 the material composition of GFRC mixtures from different reviewed studies is presented.

**Figure 2:**
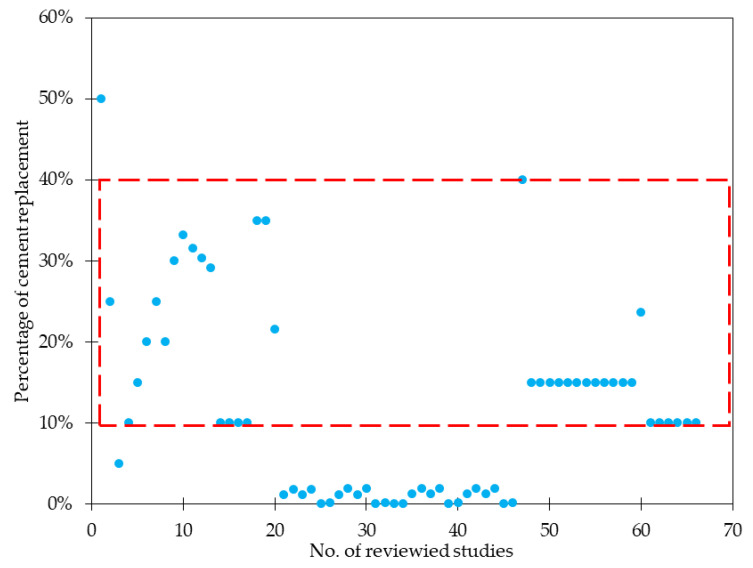
Cement replacement in studies presented in Table 3.

**Figure 3:**
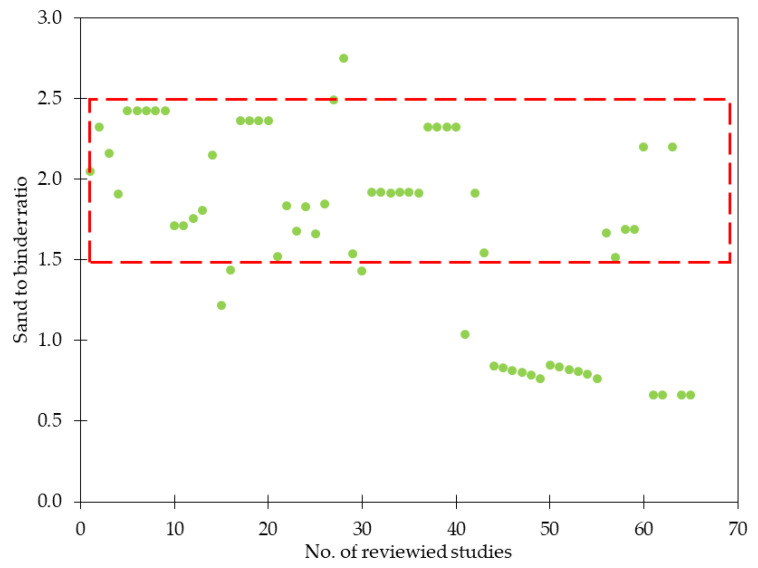
Sand to binder ratio in studies presented in Table 3.

By its analysis, it can be concluded that the water to binder ratio (w/b) usually varied between 0.35 and 0.55. However, in [35], owing to a really small w/b ratio equaled to 0.12–0.14 it was possible to obtain ultra-high performance fiber reinforced concrete with *f_c_* as high as 140–160 MPa. It is commonly known that the greater the w/b ratio, the lower the *f_c_*. As a result, depending on the requirements, the individual concrete has the appropriate amount of water, cement, aggregates, and other additions. Regarding cement, usually Ordinary Portland Cement (OPC) was used, for example, according to Eurocode standards: CEM I 42.5N, CEM I 42.5R, and following Bureau of Indian Standards: OPC 43 and OPC 53, sometimes Portland Slag Cement (PSC) and Portland Pozzolana Cement (PPC). However, to decrease the contribution of cement in concrete composition, different types of replacements were proposed and studied i.e., silica fume (SF), nano-silica (NS), fly ash (FLA), blast furnace slag (BFS), and metakaolin (MK). According to Figure 2, these materials replaced 10–40% of cement. The limitation of cement amount aims to reduce the environmental impact of the concrete industry since it is in charge of around 8–9% of total carbon dioxide in the atmosphere [39]. In the idea of sustainable development more and more emphasis is placed on decreasing this number and improving the concrete production process. On the other hand, not only the environmental footprint is erased, but the addition of other pozzolans materials leads to improved compressive, flexural, and tensile strength [18,19,32,40,41], bonding properties [42], reduced chloride and water permeability, decreased sorptivity, and free shrinkage [43,44,45]. In the analyzed papers, the maximum grain diameter of aggregates (*D_ma_*_x_) equaled to 40 mm [24], nonetheless, in most cases, it was less than 25 mm. Mostly natural river sand was used, sometimes with crushed limestone sand as a fine aggregate (FA). Considering coarse aggregate (CA), usually gravel [14], crushed granite [17,20,27,31], or crushed limestone [22,26,34] were incorporated. It must be also mentioned that there is an increasing tendency to use recycled aggregates to decrease carbon emissions and be more environmental-friendly [18,30,46,47]. In many publications, there was no information about the specific type of aggregate. From Figure 3, it was concluded that for normal strength GFRC, the sand to binder ratio (cement + SF + NS + FLA +BFS + MK + others) was usually between 1.50 and 2.50 while for ultra-high performance GFRC, around or below 1.0 like in mixes’ composition from [31,36,38]. GF had usually a length up to 25 mm, most often 6 and 12 mm (Table 3). For typical GFRC, the amount of fibers does not exceed *V_f_* = 3%. It is a result of further problems with workability, fluidity, and uniform distribution of fibers. On the other hand, in the case of spray-up mixtures, *V_f_* was usually higher and in [32] it was equal to 4 to 6%, and in [48], 4 to 5% depending on the batch. It must be noted that to ensure proper distribution of fibers within the mixture, it was necessary to add an appropriate amount of superplasticizer (SP) depending on the content of fibers like it was done in [22,30,32,35].

## 4. Description of GFRC Properties

Table 4 presents the physical and mechanical properties of GFRC mixtures and their comparison with PC from different reviewed studies.

### 4.1. Workability

**Figure 4:**
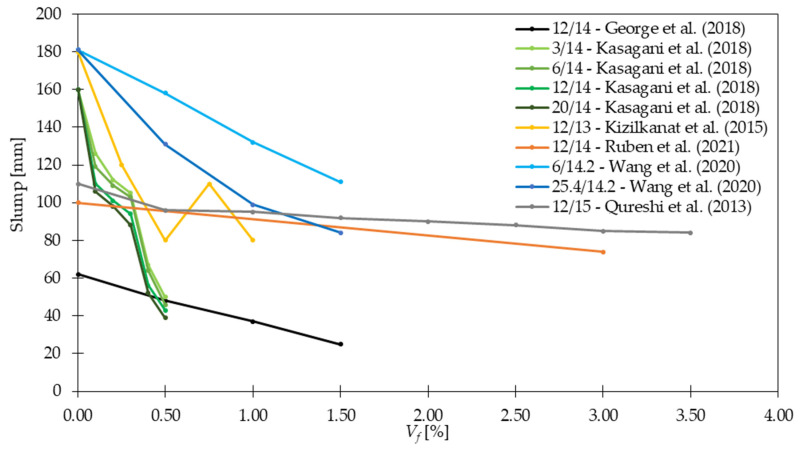
Influence of *V_f_* on concrete slump for different fiber geometry *l_f_/d_f_* [16,17,22,27,29,50].

**Figure 5:**
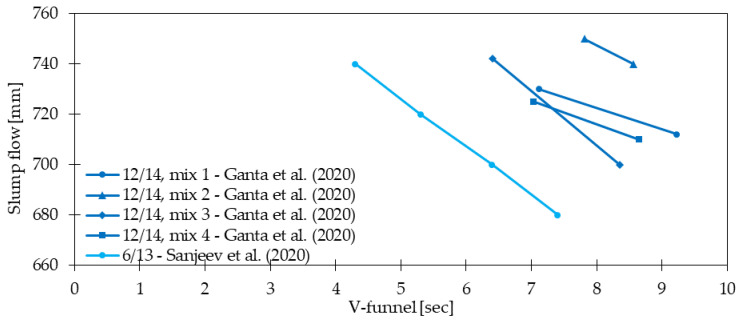
Relationship between V-funnel and slump flow for SCC for different fiber geometry *l_f_/d_f_* [21,36].

Figure 4 shows selected examples from Table 4 concerning the influence of fiber dosage on slump properties of concrete. The slump test was usually performed according to ASTM C143 [55], EN 12350-2 [56], or IS 1199 [57] where the cone was filled with concrete subjected to manual compaction. Afterward, the cone was uniformly lifted and the distance between the steel mold and the highest point of the cone was measured. On the other hand, the slump flow test and V-funnel test for self-compacting concretes (SCC) usually followed EN 12350-8 [58] and EN 12350-9 [59] standards, respectively. In the first test, the cone was filled with concrete and raised at a constant speed. Then, after the flow of the material stopped, two perpendicular diameters were measured. Regarding the second test to access the workability of SCC, the time that concrete needed to pass through the V-funnel opening was investigated. In all analyzed cases, after the addition of fibers, the fresh concrete features underwent deterioration. As a result, even for small amounts of fibers, the reduction could have reached 40% for *V_f_* = 0.05% [24], 24% for *V_f_* = 0.04% [36], and 32% for *V_f_* = 0.07% when GF reinforced mortar mixtures were used [49]. For SCC, the changes in workability were slighter. Namely, slump flow for SCC was equal to 97%, 95%, and 92% compared with PC, while slump for vibrated GFRC was equal to 87%, 82%, and 76% depending on the *V_f_* in comparison to PC [36]. Additionally, Figure 5 shows that the decrease in slump flow caused the reduction of the V-funnel. However, it must be mentioned that concrete workability is influenced not only by mix composition and fiber dosage but also by the material, shape, and slenderness of the fibers [60,61,62,63]. Based on the presented literature survey, special attention should be paid to the workability because the positive influence of fibers on different properties will be noticed as long as sufficient workability is provided. Finally, in many studies [64,65,66] the idea of an optimally designed mix in which a compromise is attained between workability and strength of the concrete mix. 

### 4.2. Crack Limitation and Shrinkage

**Figure 6:**
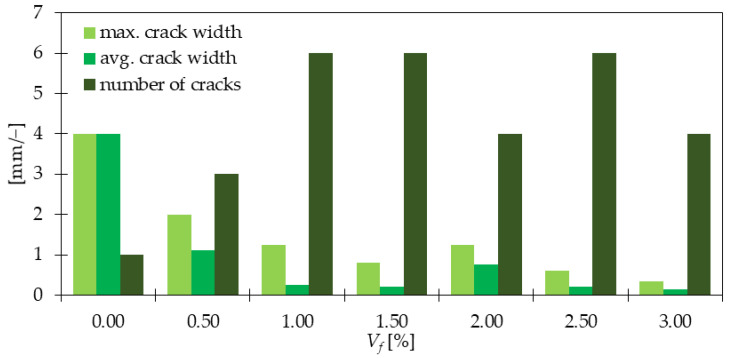
Influence of *V_f_* on the crack characterization of samples subjected to restrained shrinkage after 220 days of drying [68].

Abilities of crack bridging and shrinkage restraining are one of the most important features of fiber reinforced concretes (FRC). In [67], Patil and Burile observed that the crack width of samples subjected to the splitting tensile test (STT) underwent reduction when GF were added, especially when *V_f_* was increasing. Additionally, a comprehensive study on fiber influence was performed by Mirza and Soroushian [68]. According to them, GF were able to limit shrinkage cracking, change the failure mode into multiply cracking, as well as decrease crack width. In Figure 6, it is shown that after GF inclusion the maximum crack width decreased from around 2 to 12 times depending on the number of fibers. Similarly, regarding average crack width, the enhancements were even more significant and the best performance was obtained for the mixture with 3.0% of GF. The crack was about 0.15 mm wide which gave 27 times the smaller value compared with 4 mm for PC. The majority of the mixtures (except the one with *V_f_* = 2.0%) followed the trend that higher GF dosage led to smaller crack width. It was explained by the greater ability of crack bridging since there were more fibers. As it was mentioned before also the number of cracks increased for GFRC from one to a maximum of six cracks, changing the destruction manner. The plastic shrinkage test results performed in [69] revealed that the maximum and average crack width for GFRC was equal to 1.123 and 0.379 mm which was 57 and 50% less than PC, respectively. Furthermore, the total crack area underwent a reduction since it decreased from 253.90 for PC to 109.24 mm^2^ for GFRC, so by 57%. It was also observed that PC was characterized by a denser crack pattern while GFRC had multiple subparallel cracks. Considering different types of mixtures, GFRC results were worse than those for steel fiber reinforced concretes (SFRC) but better from polypropylene fiber reinforced concretes (PPFRC). Another research done by Barluenga and Hernández-Olivares [70] stated that the addition of 0.6 and 0.9 kg/m^3^ of GF to standard concrete type I reduced the crack area by around 60–70% depending on the type of fiber. It must be noted that according to them further increase in fiber dosage did not significantly influence this efficiency. Considering the standard concrete type II enhancements were in the range of 55 to 95%. Additionally, the crack lengths were reduced by more than 90% independently of fiber type and dosage. Considering SCC, the GF incorporation caused a 70–80% decrease in the cracked area for all the mixes. Only the batch with 0.9 kg/m^3^ of Cem-fil Anticrack HD type was characterized by less significant improvement. Similar to standard concrete, maximum crack lengths were measured for SCC and it occurred that they equaled 35–65% of maximum crack lengths for SCC without fibers. Messan et al. [71] also researched the influence of GF on the shrinkage of mortar. They reported a significant reduction of free early age shrinkage which was explained by good bonding between GF and cementitious matrix. Furthermore, it was stated that the GFRC shrinkage had a more homogenous nature than that for mortar without fibers. Finally, the positive role of GF in restraining cracks and in limiting the risk of their occurrence was concluded. Regarding restrained shrinkage, in [72] it was claimed that 1% of GF decreased the restrained stresses in mortar by 24% at 24 h. Moreover, GF were more beneficial than synthetic and metallic fiber in the reduction of the restrained shrinkage. Malathy et al. [73] studied the influence of different dosages of GF on restrained plastic shrinkage of concrete with SF. They claimed their efficiency in crack limitation even for relatively small dosages of GF like 0.3%. Additionally, in the article, it was highlighted that when SF was used, the addition of GF was necessary to arrest the shrinkage cracks. In [74], it was proven that the autogenous shrinkage was controlled when GF were added to the mortar mixture. For mixtures cured for 28 days with 2.5 and 5% of GF, the autogenous shrinkage decreased by 6.7 and 16.5%, respectively compared with the mixture without fibers. Additionally, GF had a similar influence on the drying shrinkage of samples. Namely, according to Soranakom et al. [75], even a small amount of GF equaled to 3 kg/m^3^ was able to delay the time of crack occurring from 3.5 days for PC to 4.5 days for GFRC. Additionally, the crack width decreased from 1.44 for the control sample to 0.42 mm for the specimen with GF. In summary, the addition of GF has a favorable influence on the limitation of cracks’ widths and their total area. Furthermore, the different types of shrinkages are effectively restrained by the presence of GF. It also must be mentioned that plenty of research was performed to investigate the positive influence of fibers on crack limitation and shrinkage. However, there is a limited number of works that focus on GF and not on SF, PPF, and basalt fibers (BF).

### 4.3. Mechanical Properties

#### 4.3.1. Modulus of Elasticity

**Figure 7:**
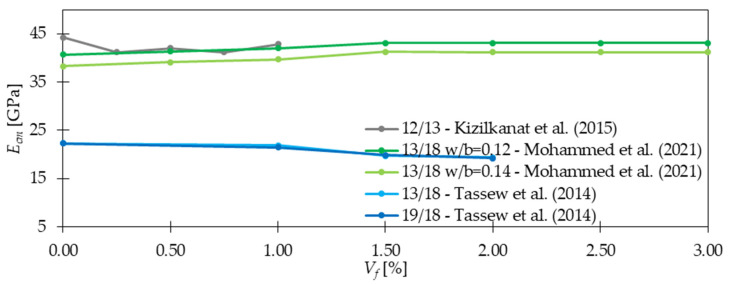
Influence of *V_f_* on *E_cm_* for different fiber geometry *l_f_/d_f_* [22,35,54].

Mohammed et al. [35] researched the mechanical properties of ultra-high performance fiber reinforced concretes with GF and in Figure 7, the results of their studies are presented. The tested samples had a cubic shape of dimensions 150 × 150 × 150 mm following EN 1352 [76]. According to them, the increase of fiber dosage improved *E_cm_* up to *V_f_* = 1.5%, then there was no significant change in the modulus. Based on these findings, it can be indicated that the presence of fibers positively influenced the stiffness of concrete, since microfibers delayed the propagation of microcracks. However, when their amount in the mix was too high, it caused the deterioration of packing and bonding properties between aggregates, fibers, and matrix. Finally, it was concluded that the optimization process is necessary to ensure proper fiber distribution. Regarding smaller fiber dosages in mortars like in [49], where *V_f_* was in the range 0.02–0.07%, the effect of fibers was insignificant, namely, *E_cm_* increased by up to 2% for the highest fiber dosage. Kizilkanat et al. [22] measured *E_cm_* following ASTM C469 [77] on cylindrical specimens with dimensions of 100/200 mm. While studying GF and BF influence on the concrete mixture, it was concluded that *E_cm_* slightly reduced when fiber dosage increased. Namely for PC, *E_cm_* was equal to 44,305 MPa and for concrete with GF and BF in the range of 41,237–42,831 MPa and 41,860–44,415, respectively depending on *V_f_*. The maximum drop of 7% was noted for the GFRC mix with 0.25% of fibers. Additionally, it was observed that when GF were incorporated, *E_cm_* was slightly lower than that for BF, which can be a result of a smaller modulus of elasticity of GF compared with BF. On the contrary, in [34] 13% growth of *E_cm_* was seen when 2.5% of GF (*l_f_/d_f_* = 6 + 18 mm/13μm) were added to the batch. *E_cm_* was measured following standard ASTM C469 [77], 150 mm in diameter and 300 mm in height samples were tested. In [54], for the mixture where fine and coarse LECA was used as an aggregate, a small increase of *E_cm_* was noted, up to 6% when 1.0 and 2.0% of GF were incorporated. On the other hand, when only sand was used without any coarse fraction, for *V_f_* = 2%, the maximum reduction equaled to 14%. Tassew and Lubell [54] also used indications of ASTM C469 [77] to test *E_cm_* on 100 mm diameter × 200 mm height cylindrical specimens. Based on the presented research, it was concluded that the effect of GF on *E_cm_* is ambiguous. Namely, they may increase the stiffness of concrete when uniform fiber distribution is provided, however, their negative effect on *E_cm_* is also recognized in the literature.

#### 4.3.2. Compressive Strength

**Figure 8:**
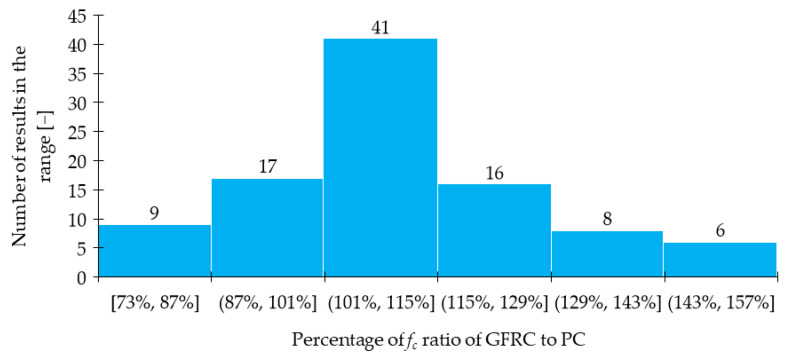
Histogram of *f_c_* ratio of GFRC to PC for the studies reviewed in Table 4.

**Figure 9:**
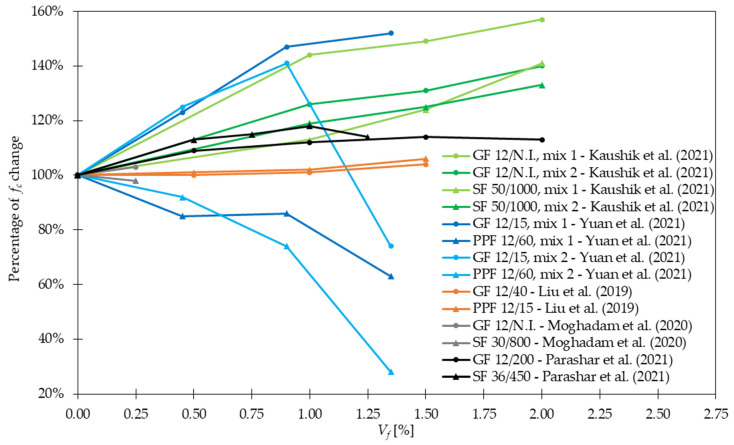
Change of *f_c_* in relation to *V_f_* for different fiber material and geometry *l_f_/d_f_* [19,23,26,33,53].

Based on the findings reported in Table 4, the histogram of *f_c_* change was prepared (Figure 8). It can be concluded that most of the analyzed cases were in the range of 101 to 115% because as many as 41 out of 97 examples were included in it. Additionally, around three-fourth of all results experienced an increase of *f_c_*. The fall of this strength for some mixtures can be attributed to the workability deterioration like in [29], where the slump dropped from 181 mm to 111 mm and 84 mm after the addition of 1.5% of *l_f_/d_f_* = 6 mm/14.2 μm and *l_f_/d_f_* = 25.4 mm/14.2 μm GF, respectively leading to the reduction of *f_c_*. Similar dependencies were recognized for glass fiber reinforced mortars. Namely, in [78] the compressive strength increased from 73 MPa for mixtures without fibers to 96 MPa for mix with 2.5% of GF and afterward dropped to 87 MPa for *V_f_* = 3.5%. The problem of an excessive amount of fibers which could have caused nonuniform fiber distribution and as a result *f_c_* decrease was also visible in the following studies [15,25,32,50,51]. Additionally, Abdollahnejad et al. [79] stated that with the increase in fiber length, the reduction of *f_c_* was greater. Regarding mortars, in [78] the early age strength of mixture with GF exhibited higher values than plain mortars. On the other hand, the early development rate of compressive strength was decreased. Findings by the other authors presented in Figure 9 have also shown that for the same *V_f_*, GFRC behaved comparable or in many cases significantly better than SFRC or PPFRC. In conclusion, a higher percentage improvement of *f_c_* can be obtained when GF are added to the mixture than SF or PPF. Furthermore, in many cases, the positive influence of simultaneous incorporation of two types of fibers (GF + SF or GF + PPF) instead of one was highlighted [21,26].

#### 4.3.3. Tensile Strength

When designing concrete elements, knowledge about the tensile behavior of the material is required. In the uniaxial tensile test (UTT), the tensile strength (*f_t_*) results directly from the data received during the experiment. However, UTT is a very demanding test and has to be performed on the ideal sample under well-controlled conditions that eliminate any eccentricities. For these reasons, UTT is seldomly used in research practice [80]. More often flexural tensile test (FTT) is proposed i.e., in many standards such as Model Code 2010 [81] and Technical Report 34 [82] or in numerous published articles [83,84]. Usually, a three-point bending test (3PBT) with a notch [85,86] and a four-point bending test (4PBT) without a notch [87,88] are performed to calculate the flexural tensile strength (*f_fl_*). This test is used willingly because it is easier to execute and less time-consuming. Nonetheless, a significant amount of elastic energy stored in the prism specimen should not be neglected. Additionally, it must be mentioned that through this method only the indirect determination of tensile strength is possible. To reduce even more the complexity of the tensile characterization, STT was developed. Namely, the samples are smaller, hence easier to transport and the test is more stable. The splitting tensile strength (*f_spl_*) received from STT also requires some additional calculations because it is an indirect method of tensile strength characterization.

**Figure 10:**
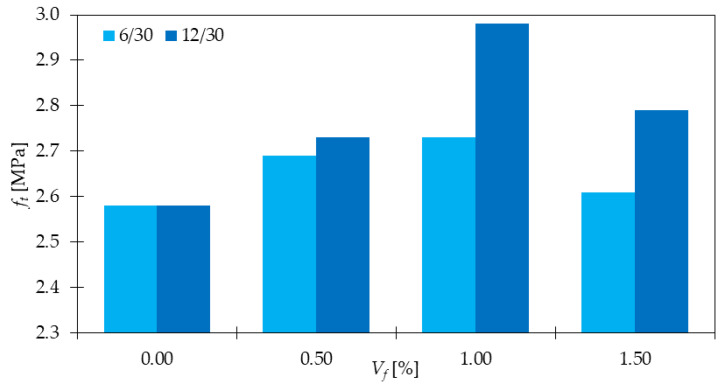
Influence of *V_f_* on *f_t_* for different fiber geometry *l_f_/d_f_* [14].

**Figure 11:**
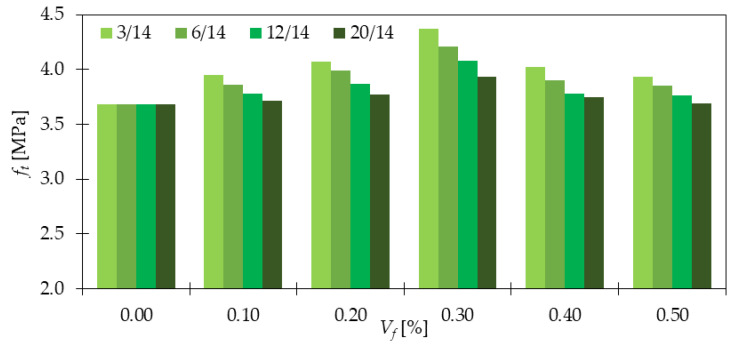
Influence of *V_f_* on *f_t_* for different fiber geometry *l_f_/d_f_* [17].

Regarding UTT, the clear influence of GF inclusion was visible in [14]. Namely, after addition of 0.5, 1.0, and 1.5% of *l_f_/d_f_* = 6 mm/30 μm and *l_f_/d_f_* = 12 mm/30 μm, fibers, *f_t_* increased by 4 and 6%; 6 and 16%; 1 and 8%, respectively (Figure 10). The highest enhancement was noted for longer fibers. This is contrary to the results from [17], where shorter fibers were characterized by greater improvements (Figure 11). The maximum one was denoted for a mixture with 3 mm long fibers for *V_f_* = 0.3%, while the minimum one for *l_f_* = 20 mm with 0.1 and 0.5% of GF. Nevertheless, in both articles, attention was paid to the idea of the optimum fiber dosage since more fibers did not always lead to better mixture performance. Kasagani et al. [17] also highlighted the concept of hybrid mixtures, where two (*l_f_/d_f_* = 3 + 6 mm/14 μm and *l_f_/d_f_* = 12 + 20 mm/14 μm) or four types of GF (*l_f_/d_f_* = 3 + 6 + 12 + 20 mm/14 μm) were mixed. It occurred that a combination of more than one fiber type can significantly overcome results for a mono mix for the same amounts of fibers. For example, for *l_f_/d_f_* = 3 + 6 mm/14 μm of GF (*V_f_* = 0.06 + 0.24%), *f_t_* increased by 41%, while for *l_f_/d_f_* = 3 mm/14 μm of GF (*V_f_* = 0.30%) and *l_f_/d_f_* = 6 mm/14 μm of GF (*V_f_* = 0.30%), *f_t_* was only 19 and 14% higher than that for PC, respectively. The best results from the campaign were noticed for mixture with *l_f_/d_f_* = 3 + 6 + 12 + 20 mm/14 μm of GF because the tensile strength improved 1.5 times compared to concrete without fibers.

**Figure 12:**
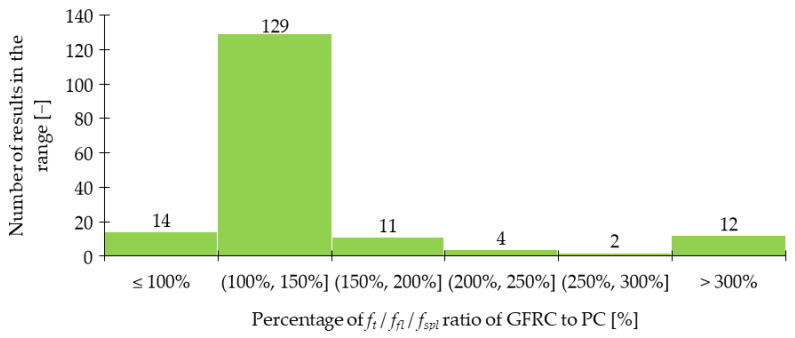
Histogram of *f_t_*/*f_fl_*/*f_spl_* ratio of GFRC to PC for the studies reviewed in Table 4.

**Figure 13:**
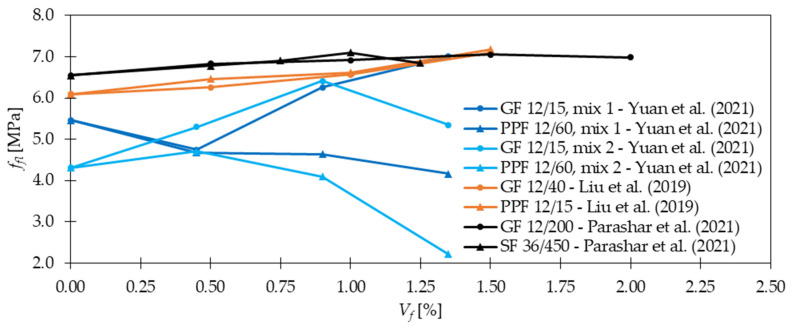
Influence of *V_f_* on *f_fl_* for different fiber material and geometry *l_f_/d_f_* [23,26,53].

**Figure 14:**
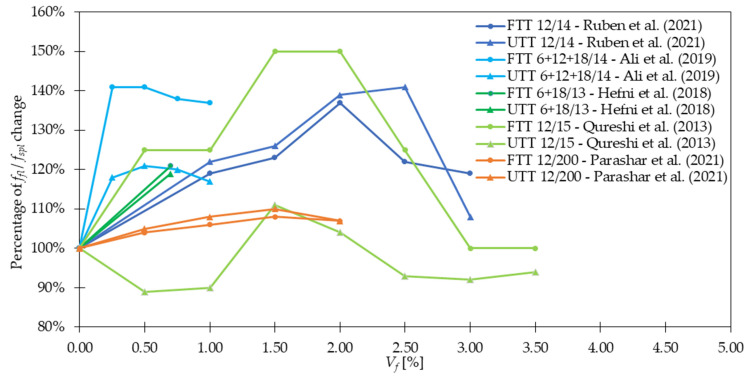
Change of *f_fl_*/*f_spl_* in relation to *V_f_* for different fiber geometry *l_f_/d_f_* [27,30,34,50,53].

**Figure 15:**
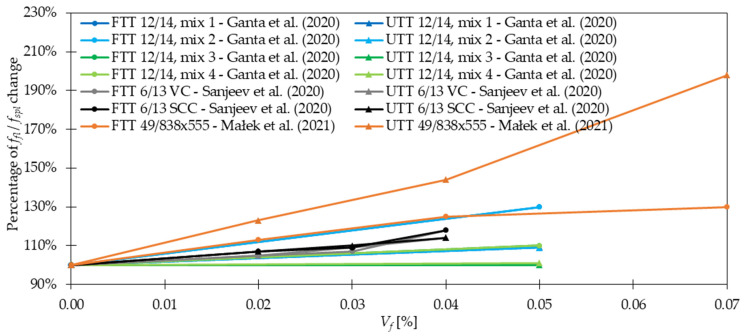
Change of *f_fl_*/*f_spl_* in relation to *V_f_* for different fiber geometry *l_f_/d_f_* [21,36,49].

Regarding *f_fl_* and *f_spl_*, in the majority of cases presented in Table 4, an increase in strength after fiber addition was visible. According to Figure 12, the improvement was usually in the range of 0 and 50%. However, it must be mentioned that incorporating more GF did not always result in significant advancement of tensile properties. In [30], the level of improvement (41%) was the same for mixtures with 0.25% and 0.50% of GF, while for concrete with 1.00% of GF, even a bit smaller (37%). Similarly in [53], increasing *V_f_* twice led to *f_fl_* change from 6.92 to only 6.98 MPa. Furthermore, based on the findings reported in Table 4, it can be concluded that for the same fiber geometry but different concrete composition, however, with more than three times smaller amounts of GF, similar improvements can be achieved. On the other hand, it must be mentioned that fiber geometry has also an influence on the results. Namely, in [79] the higher values of *f_fl_* were achieved for GF which were 30 mm long than for those which were just 10 mm long. Furthermore, in [54] it was concluded that even a small difference in fiber length from 13 to 19 mm increased *f_fl_* by not 257, 429, and 481% but by 362, 462, and 514% when 1.0, 1.5, and 2.0% of GF were added, respectively. Similar conclusions about the positive influence of longer fibers were revealed in [29]. Additionally, a decrease of *f_fl_* and *f_spl_* for greater *V_f_* is observed due to problems with workability and uniform distribution of fibers. In conclusion, the optimization of the concrete mix is a key process that allows finding the compromise between the requirements that the individual mix should fulfill and its costs. Ruben et al. [27] incorporated *l_f_/d_f_* = 12 mm/14 μm of 1.0–3.0% with a step of 0.50% of cement weight of GF to the concrete mixture. The enhancement in *f_fl_* was respectively 19, 23, 37, 22, and 19%, while in *f_spl_* 22, 26, 39, 41, and 8%. For example, in [21] for mixtures with concrete grade equaled 40 and 80 MPa, *f_fl_* increased 1.3 and 1.1 times when only 1.3 kg/m^3^ of GF were included, respectively. In [26], the positive impact of *V_f_* increase was acknowledged since, after the addition of 0.50%, 1.00%, and 1.50% of GF, *f_fl_* rose from 6.09 MPa to 6.26, 6.57, and 7.10 MPa, respectively. Nevertheless, in some works, even higher increases in strength when adding GF were seen; in [32], *f_fl_* was almost four times greater than that for concrete without fibers; in [38,51] the maximum force in flexural test grew twice; while in [54] the failure load for GFRC was more than six times higher than that of PC. In the case of mortars, according to [89], more than 10% improvement was noted when *f_fl_* changed from 7.31 for the plain mixture to 8.21 MPa for mixture with GF, and from 7.26 to 8.02 MPa for w/b = 0.34 and w/b = 0.40, respectively. In [78], it was possible to obtain even higher values of flexural strength of mortar equaled to 17.2 MPa so 67% greater than that of the sample without fibers (10.3 MPa). Moreover in [21], a combination of 0.05% of GF and 1.0% of SF exceeded improvements for mixtures with just one type of fiber leading to the conclusion that hybrid mixtures can more effectively influence the strength. The hybridization advantages were also emphasized by Liu et al. [26], where replacing half of GF with PPF caused strengthening of concrete by 8, 19, and 20% for GF + PPF = 0.25 + 0.25%, GF + PPF = 0.50 + 0.50%, and GF + PPF = 0.75 + 0.75% in comparison to PC, respectively. It must be mentioned that all these results were greater than the ones for batches where only GF or PPF were used. In Figure 13, the effectiveness of GF in comparison to fibers from other materials like SF and PPF is presented. In the case of [23], the superior behavior of GF was visible, while in [26,53] the benefits from GF and PPF or SF incorporation were quite similar. However, attention should be paid because the geometries of fibers were different. Kizilkanat et al. [22] used BF and GF of the same length 13 mm, and similar diameter. The results from the experiment revealed that in the majority of cases the behavior of GFRC both in STT and 3PBT was more beneficial. Additionally, the maximum enhancement was equal to 27% for *f_spl_* when *V_f_* = 0.75% and 32% for *f_fl_* when *V_f_* = 0.50% of GF were added. However, beyond *V_f_* = 0.75% the dominant improvement for basalt fiber reinforced concretes (BFRC) was concluded which could have been attributed to the difficulties in GF dispersion in the concrete matrix. In the end, the comparison of enhancements after GF addition in FTT and STT is shown in Figure 14 for larger *V_f_* and in Figure 15 for smaller *V_f_*. It was concluded that usually, the percentage change of *f_fl_* and *f_spl_* was quite similar [27,34,36,53] or flexural test gave higher enhancement levels [21,30,50], seldomly the splitting of the sample resulted in a rise bigger than that from FTT [49].

### 4.4. Post-Cracking Behavior, Ductility, and Toughness

**Figure 16:**
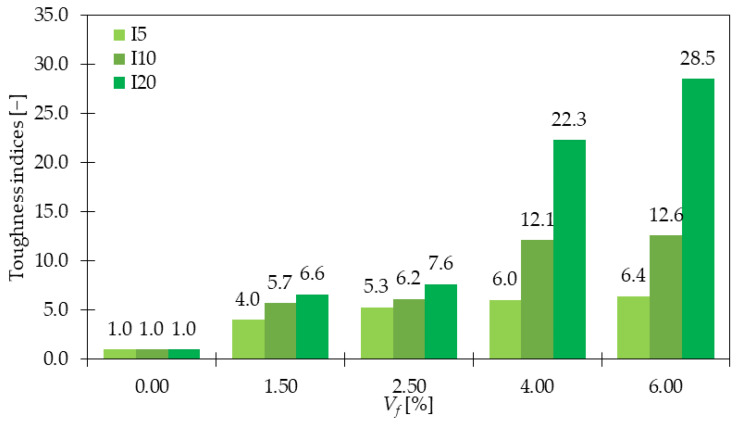
Toughness indices: *I_5_*, *I_10_*, and *I_20_* in relation to *V_f_* [32].

**Figure 17:**
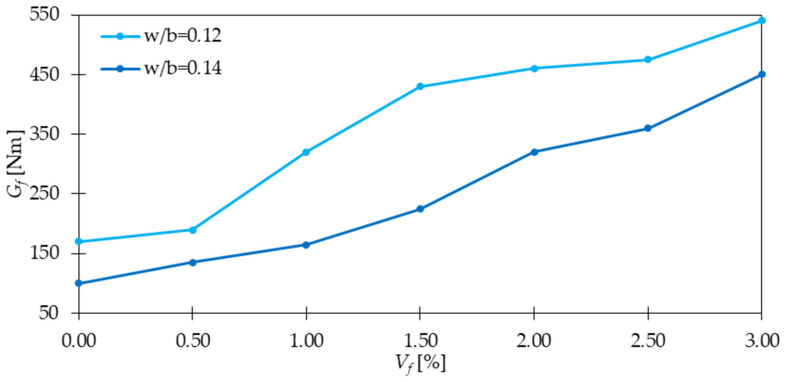
Fracture energy *G_f_* in relation to *V_f_* [35].

When studying the post-cracking behavior of concrete, it is important to analyze the fracture energy (*G_f_*), which is the energy required to break the sample, and fracture toughness (*K_IC_*) which is defined as the ability of the specimen to resist brittle failure or crack extension [22]. In the work [22], fracture energy increased by 40, 46, and 64% for samples with 0.50, 0.75, and 1.00% of GF, respectively when compared with PC. Additionally, the fracture toughness was around 1.37 and 1.75 times higher for GFRC with 0.50 and 1.00% of GF, respectively than that for PC. Findings that GF have a remarkably positive impact on concrete ductility were also recognized in the work of Morteza, et al. [32]. From the toughness indices calculation according to ASTM C1018 [90], it was concluded that with the increase in fiber content, the indices *I_5_*, *I_10_*, and *I_20_* also rose (Figure 16). Moreover, for higher GF dosages the differences between individual indices were greater. Additionally, these significant toughness improvements meant that with the increase of the deflection, the sample was still able to resist significant forces. For all specimens with fibers, the ductile behavior was noticed, in contrast to the plain samples, which underwent brittle failure. In [35], regardless of w/b, the fracture energy improved after the addition of GF. From Figure 17 it can be concluded that adding 3% of fibers led to 3.1 for w/b = 0.12 and 4.7 for w/b = 0.14 times greater *G_f_*. Mentioned conclusions are in agreement with the findings of [38,54], where additionally it was observed that lightweight concretes had lower values of flexural toughness than standard concretes. This was explained by the preference of crack occurrence not only through the binder but also through the lightweight aggregate. To sum up, the significant improvement in the post-cracking behavior, ductility, and toughness due to the inclusion of GF is the result of fiber ability of crack bridging and energy-absorbing. The enhancement is usually higher for greater *V_f_* since a higher number of fibers can arrest more cracks. Additionally, more GF means stiffer concrete with higher improvements in tensile strength and as a result, harder material to break. Considering this, higher energy is necessary to fracture GFRC than the PC specimen.

### 4.5. Impact Resistance

**Figure 18:**
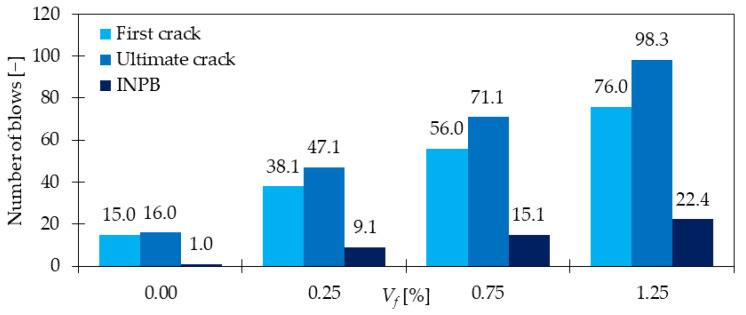
Impact resistance in relation to *V_f_* [91].

**Figure 19:**
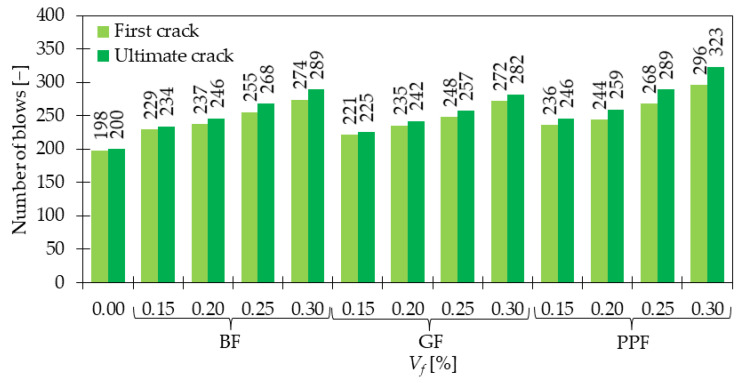
Impact resistance in relation to *V_f_* for different types of fibers [94].

The impact resistance of GFRC is significantly improved compared to PC. Mastali et al. in [91] tested the property according to ACI Committee 544 [92]. They dropped the steel hammer of 4.45 kg from a height equaled to 457 mm on a steel ball. The diameter of the ball was 63.5 mm and it was in contact with the concrete disc sample of diameter 150 mm and a height of 65 mm. The number of blows to cause the first visible crack and final failure of the sample was calculated. It was revealed that the number of blows for the first crack increased 2.53, 2.37, and 5.06 times when subsequently 0.25, 0.75, and 1.25% of GF were added. Regarding ultimate failure, the value of blows rose 2.94, 4.44, and 6.14 times. Additionally, the number of post-initial crack blows to failure which was called INPB was calculated and its measures were equal to 9.00, 15.08, and 22.35 times more than that for PC, depending on *V_f_*. The results of the study [91] are presented in Figure 18. Additionally, it was concluded that higher amounts of GF led to better improvements. Namely, with the increase of *V_f,_* the material was stiffer and had higher tensile strength which resulted in greater difficulties in cracking. Furthermore, the difference between failure modes was visible, namely, for a higher number of fibers, multiple cracks occurred on the specimen surface, due to the bridging effect. The result in [93] also supported the idea of the positive influence of GF on impact resistance measured in accordance with ACI Committee 544 [92]. Both impact energy at first and ultimate crack were improved by around 2, 98, 179, and 252% for concrete grade (CG) 30 and 27, 34, 68, and 97% for CG 40 for mixes with respectively 0.5, 1.0, 1.5, and 2.0% of GF (*l_f_/d_f_* = 12 mm/14 μm). It is noteworthy, that higher enhancements were visible for a smaller concrete grade, however, values of impact energies were greater for concrete with higher compressive strength. Finally, in [94] the influence of fibers from different materials such as basalt, glass, and polypropylene was analyzed. From Figure 19 it was concluded that the addition of any type of fiber increased the impact strength. The energy required to create first crack for *V_f_* = 0.00, 0.15, 0.20, 0.25, and 0.30% of GF was equal to 3950, 4409, 4688, 4948, and 5426 J, subsequently. On the other hand, the failure impact energy was 3990, 4489, 4828, 5127, 5626 J. The impact energy calculations required knowledge about the mass and the fall height of the hammer and the number of blows. The test was performed in accordance with [92]. Similarly to [91], for GFRC with 0.30% of fibers, the crack pattern changed from one failure crack to a multiple crack number. Finally, Yildirim et al. [95] summarized that even for a small dosage of GF like 2.68 kg/m^3^ (*V_f_* = 0.1%) the impact resistance can be remarkably strengthened. Furthermore, the hybrid FRC combining SF and GF significantly outperformed the behavior of mixtures with only one type of fiber. It must be mentioned that in the presented research, the hybrid blend with steel and glass fibers was usually more effective than the one with steel and polypropylene fibers. The beneficial aspect of hybridization was macro SF arrested macrocracks while micro GF and PPF bridged microcracks.

### 4.6. Spalling and Fire Resistance

**Figure 20:**
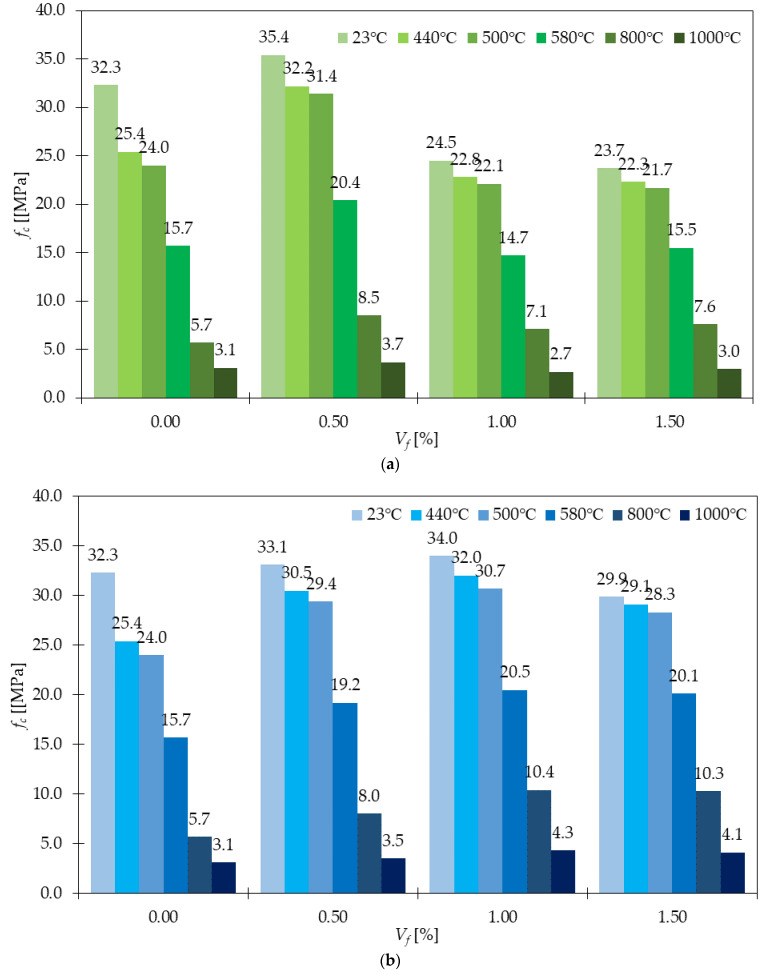
Effect of high temperature on *f_c_* of PC and GFRC for GF with different lengths: (**a**) *l_f_* = 6 mm; (**b**) *l_f_* = 25.4 mm [29].

In the concrete matrix, water can be found in the form of free water and bound water. When hardened concrete is subjected to high temperatures, the water inside the concrete evaporates and generates internal tensile pressure if entrapped vapor does not have the path to escape. The spalling of concrete occurs due to this phenomenon. In GFRC, the capacity to resist extreme temperatures is increased because the tensile strength is improved. This is in agreement with the findings of Mirza and Soroushian described in [68], where *f_fl_* of samples with GF after exposure to high temperature decreased, however, these reductions were smaller than the ones for PC. The effectiveness of GFRC in enhancing the spalling resistance is also explained by GFs’ ability of crack arresting: limiting their number, width, but also changing the form of failure from one macrocrack to multiply microcracks. The effect of high temperatures (440, 500, 580, 800, and 1000 °C) on *f_c_* losses was also investigated by Wang et al. in [29] and the results of the studies are presented in Figure 20. The cylindrical specimens were placed in the electronic furnace for a heating process for 48, 54, 60, 105, and 160 min, respectively depending on the temperature. According to the researchers’ conclusions, the residual compressive capacity rose with the GF dosage and fiber length. An important conclusion was also made in the study [96] where fibers’ addition eliminated the explosive spalling of concrete samples subjected to 100, 200, 300, 400, 500, 600, 700, 800 °C. The rate of temperature rise was 240 °C/h and after reaching the target temperature, samples were kept in it for 2 h. The purpose of this regime was to obtain the homogenous temperature within specimens. It was revealed that the behavior of GFRC for different temperatures always outperformed PC. Nevertheless, for all mixtures *f_c_*, *f_fl_*, and *f_spl_* increased up to 300 °C and then started to reduce. Additionally, after reaching 500 °C the degradation changes were more visible in the case of concrete without fibers compared with FRC. Finally, it was stated that the mixture with optimized performance regarding compressive, flexural, and splitting characterization subjected to normal and high temperatures was a hybrid mixture of 2% of SF + 1% of GF. This again claimed the advantageous side of hybrid concretes. The results presented in [33] also supported the idea of improved FRC fire resistance. For samples subjected to the ambient temperature: 100, 200, 300, 350, 400, 450, 500, 650, and 800 °C, *f_c_* increased by 9–27% and 0.06–18.40% for SFRC and GFRC respectively; the *f_spl_* increased by 7.96–197.74% and 19.22–213.53% for SFRC and GFRC respectively compared to PC. It was noted that GF were more effective in tensile tests than SF, in contrast to the compression test. Additionally, the weight loss of samples exposed to elevated temperatures was smaller for FRC than for PC. In the end, it is noteworthy to mention that GFRC permanent formworks can upgrade fire protection. Namely, in [97] it was stated that columns in such formworks had an extra hour fire rating compared to columns made with the usage of removable timber formworks.

### 4.7. Abrasion Resistance

**Figure 21:**
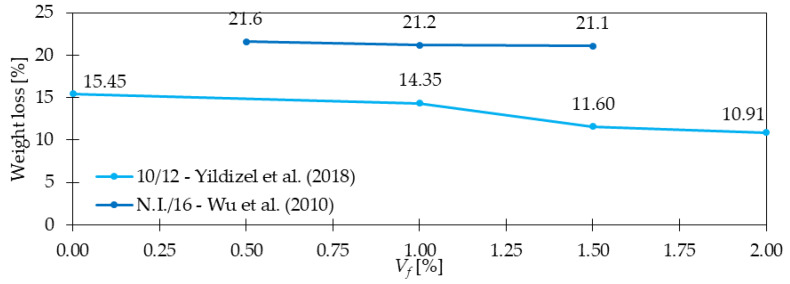
Weight loss in relation to *V_f_* for different fiber geometry *l_f_/d_f_* in flow abrasion test [98,100].

**Figure 22:**
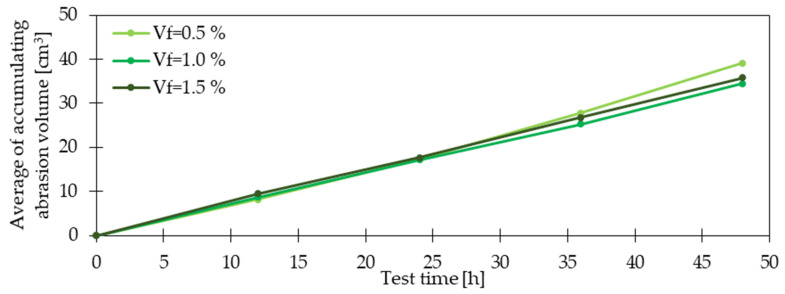
Average of accumulating abrasion volume in relation to the testing time of underwater abrasion resistance test [100].

In the study [98], the abrasion resistance was determined in a sand blasting process following ASTM C944 [99]. It was researched that the weight loss during abrasion testing was greater for concretes without fibers than for GFRC. For *V_f_* = 2.0% (*l_f_/d_f_* = 10 mm/12 μm) the loss in weight for the sand blasting process was almost 5% smaller compared with PC (Figure 21). Wu et al. [100] in their experimental program contained a flow abrasion test and underwater abrasion resistance test on GFRC. In the first one, the sample’s surface was subjected to the impact of waterborne sand which was pumped through the specially designed nozzle. The flow of waterborne sand on the specimen with dimensions 200 × 200 × 50 mm was kept at 12 m/s and maintained for 2 h. The abrasion erosion resistance was calculated by weight reduction of the sample. The second method called the abrasion resistance test of concrete underwater method was performed according to ASTM C1138 [101]. The concrete sample with 300 mm in diameter × 100 mm in height was placed at the bottom of the steel tank filled with water. On the specimen, steel grinding balls were situated. The agitation paddle was rotating at a speed of 1200 rpm. During the test, the sample was weighted every 12 h for a 2-days test period. The abrasion erosion resistance was measured by weight reduction of the sample. The results of the first abrasion test are presented in Figure 21 and the outcomes of the second testing method in Figure 22. From both of them, it can be concluded that the GF addition had a positive influence on the abrasion resistance of concrete. However, attention should be paid to the number of included fibers since too high dosages may lead to opposite effects. Finally, according to the authors’ knowledge, there is a limited number of articles considering the role of GF in abrasion resistance in contrary to the influence of PPF and SF on this property [102,103,104].

### 4.8. Thermal Conductivity

**Figure 23:**
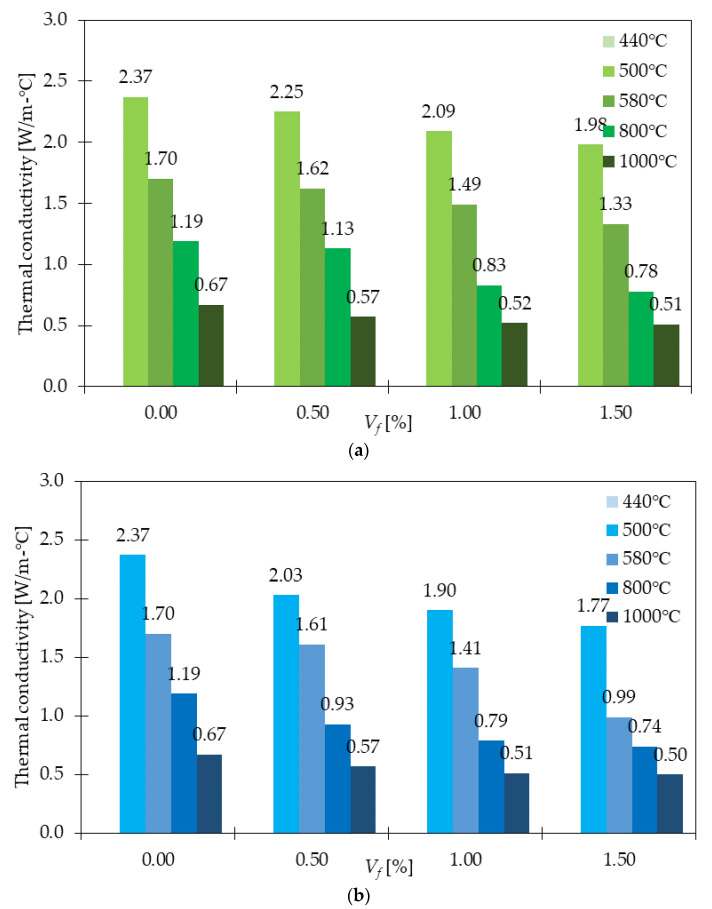
Thermal conductivity in relation to *V_f_* for GF with different lengths: (**a**) *l_f_* = 6 mm; (**b**) *l_f_* = 25.4 mm [29].

Concrete with the addition of GF have lower thermal conductivity than SFRC [105] and carbon fiber reinforced concretes (CFRC) [106]. It was proved by the tests performed in [105]. First, cube specimens were stored in wet conditions and then in a climatic chamber for 7 and 21 days, respectively. Afterward, the samples were framed with styrofoam stocks. Then, one-dimensional steady-state heat flow was introduced and the samples were subjected to three temperature stages. The measurements revealed that the average thermal conductivity of GFRC was equal to 2.67 W/mK (1 kg/m^3^ of GF), while 2.86, 2.85, and 2.97 W/mK when 35.0, 27.5, and 20 kg/m^3^ of SF were incorporated. Additionally, for PC the thermal conductivity had the value of 2.83 W/mK which was still higher than the value for concrete with GF. Moreover, increasing the amount of GF from 0.4 to 1.2% reduced the thermal conductivity by around 15% in [94]. A comprehensive study on thermal properties of different types of fibers: PF, SF, and GF was done by Małek et al. in [107]. The lowest thermal conductivity was noted for 1.0% of GF and the reduction in values was equal to 12% compared to PC. On the other hand, the highest increase was reported for the sample with 1.0% of SF, where thermal conductivity changed by 5.3% from 1.88 for PC to 1.98 W/mK for SFRC. The authors of the study calculated also thermal diffusivity and specific heat. It was concluded that GFRC revealed the highest thermal diffusivity compared to PPFRC and SFRC. Regarding specific heat, the decreasing trend was observed with the addition of fiber and further increase of their amount. Specific heat values for GFRC were in the range of 1.46–1.39 MJ/m^3^ K while for PC it was equal to 1.98 MJ/m^3^K. The positive behavior of GF on the thermal conductivity of GFRC under high temperatures was also discussed in [29]. Herein, both the effect of adding GF, increasing fiber dosage, and changing fibers geometry were investigated (Figure 23). First of all, it was stated that with the addition of fibers, the thermal conductivity decreased up to 16 and 25% for 6 and 25.4 mm long fibers with *V_f_* = 1.5% subjected to 440 °C, respectively. It must be mentioned, that even higher enhancements were concluded, for example for 500 and 580 °C. Second, with the increase of *V_f_*, the thermal conductivity decreased. Finally, greater improvements and smaller thermal conductivities for all temperatures were noted for longer fibers. However, for higher temperatures, the difference between short and long GF was getting less significant. All of this led to the conclusion that the addition of GF and applying GFRC instead of concrete with steel fibers can reduce the house heating energy demand which is in agreement with the concept of smart manufacturing and smart building. To sum up, GFRC are characterized by better performance from the building’s point of view.

### 4.9. Durability

#### 4.9.1. Permeability

**Figure 24:**
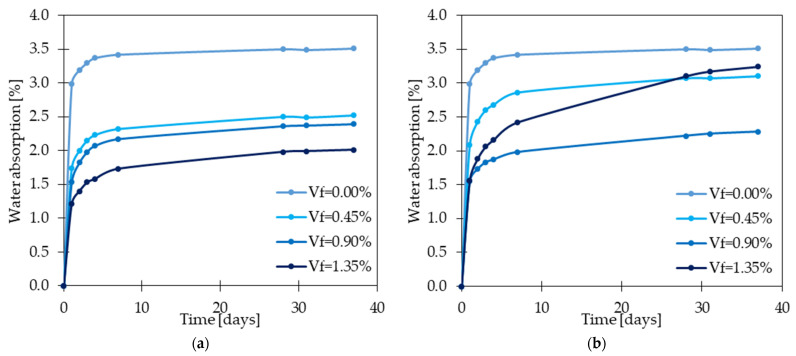
Water absorption of GFRC: (**a**) w/b = 0.30; (**b**) w/b = 0.35 [23].

The absorption, porosity, and permeability of concrete indicate the possibilities of water ingress into the concrete. Additionally, water can transport harmful agents—different types of salts which can negatively influence the durability and service life of the concrete elements. In [21], the water absorption was slightly higher for GFRC than for SFRC and hybrid fiber reinforced concretes (HFRC). On the other hand, concrete with GF had the highest ability to lose water which is called water desorption. Both tests: water absorption and desorption were performed on cubic samples of dimensions 100 × 100 × 100 mm according to ASTM C642 [108]. Namely in the first test, specimens were subjected to drying at 105 °C, then weighted and cooled. Afterward, they were immersed in water and again weighted in time intervals up to 72 h. Considering the water desorption experiment, the samples’ weights were measured before and during drying. At the same time, finding by the other authors [23] have proved that with the increase in fiber dosage, a decrease in water absorption, which was tested according to [108], was noted (Figure 24). For instance, after adding to the mixture 1.5% of GF, the water absorption changed from 3.49 to 1.99%, which meant a 43% reduction. In the article, it was also stated that GFRC behaved significantly better than PPFRC, which usually caused an increase in water absorption. Ruben et al. [27] also concluded that the water absorption, measured according to the Bureau of Indian Standard [109], decreased with the increase of *V_f_* up to a certain value. The property was equal to 4.85, 3.66, 3.24, 2.69, 2.10, and 2.74% for GFRC with 0.0, 1.0, 1.5, 2.0, 2.5, and 3.0% of GF, subsequently. Based on this data, it was calculated that the water absorption compared with PC was reduced by 24.5, 33.2, 44.5, 56.7, and 43.5%, respectively. The positive effect of fibers on water absorption could be attributed to the good bonding between the matrix and the fibers as well as the smaller permeability of concrete. This feature is also connected with concrete porosity, which in [28] for GFRC with 1.0, 2.0, and 3.0% of GF (*l_f_/d_f_* = 25 mm/15 μm) corresponded to 91, 83, and 93% compared with concrete without fibers. Furthermore, the volume of pores was smaller for GFRC than for PC. Within the porosity test, the samples were dried at 100 °C for 2 days. Afterward, the volume of the pores was estimated based on the difference between the sample’s weight before and after putting it in the hot oven. However, it cannot be said that fibers always have a positive influence on concrete permeability since in [30], the porosity and sorptivity increase was noted after fiber addition. Moreover, higher *V_f_* meant greater values of these concrete properties. The concrete porosity was tested according to ASTM C642 [108] and sorptivity following ASTM C1585 [110]. Authors explained this phenomenon by a nonuniform distribution of fibers that could have formed bundles and the possibility of connection voids and channels which all could have led to an increase in concrete permeability. This could have been also caused the increase of air content in concrete or mortar like it was described in [49]. Dehghan et al. [37] in their research highlighted the usage possibility of recycled GF. As part of the study, they analyzed the effect of different types of fibers on air content. It was concluded that for *V_f_* = 1.2% of recycled and virgin GF, with the lengths 21.7, 17.2, 21.2, 19.5, and 25.4 mm, the air content rose by 50, 12, 4, 12, and 65%, respectively. It is noteworthy, that the virgin GF had the highest air content, which showed that using recycled fibers can be a beneficial and reasonable option not only regarding sustainable development [111]. The sorptivity test of GFRC exposed to elevated temperatures was also performed within Moghadam et al. study [33]. The concrete cubes were sealed up to a height equal to 30 mm to force the bottom surface to absorb water. Additionally, 10 mm diameter bars were situated under the samples to provide free movement of water under them. The weighting process of samples took place before and during the test at the intervals of 3, 6, 24, 48, and 72 h. Herein, it was concluded that the GF addition decreased the sorptivity coefficient regarding all tested temperatures. Namely, for temperatures below 500 °C and of 650 °C the coefficient was improved by an average 37%, and for temperatures of 500 °C and 800 °C by an average 40% for GFRC. This beneficial impact was explained by the denser structure of concrete owing to the fibers. The conclusion was also supported by the water penetration depth test which equaled 6.55 mm and 4.42 mm at 100 °C for PC and GFRC, respectively. Moreover, for temperatures below 500 °C and of 650 °C, the penetration depth decreased by around 28% for GFRC in comparison to PC. The smallest penetration depth was obtained for GFRC at 500 °C since it equaled only 58% of the value for PC. However, the greatest enhancement belonged to the GFRC sample subjected to 300 °C since the penetration depth decreased by 45%. To sum up, FRC permeability results are an individual case, requiring each time experimental testing, and an unambiguous conclusion cannot be made. Furthermore, attention should be paid to the workability properties and ensuring proper fiber distribution in the concrete.

#### 4.9.2. Freeze-Thaw Resistance

**Figure 25:**
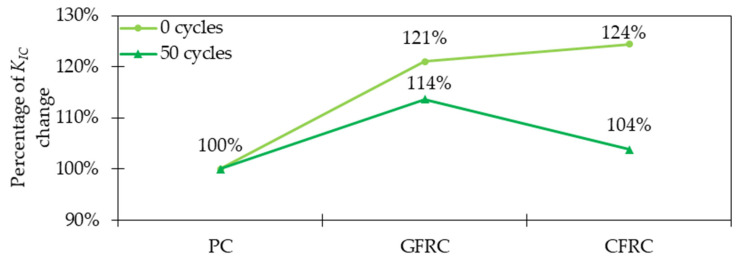
Change of *K_IC_* for PC, GFRC, and CFRC subjected to freeze-thaw cycles [113].

**Figure 26:**
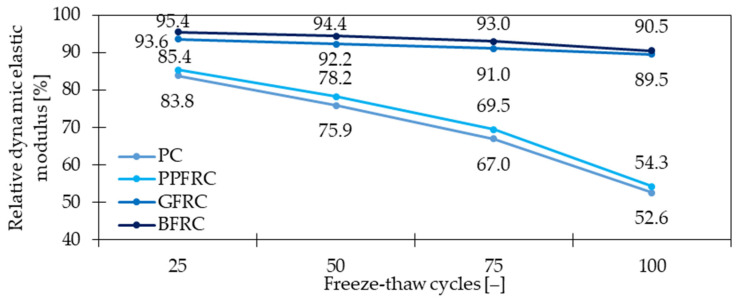
Relative dynamic elastic modulus for different FRC subjected to freeze-thaw cycles [115].

The freeze-thaw resistance can be significantly improved by the addition of fibers e.g., in face slab concrete [112]. Reis and Ferreira [113] claimed that the critical stress intensity factor *K_IC_* for FRC surpassed the *K_IC_* for PC under normal conditions. Additionally, as a result of 50 freeze-thaw cycles, where temperature changed from +20 to +100 °C, the behavior of concrete with GF was better than the one without fibers (Figure 25). This is in agreement with the findings of [114], where samples were subjected to 30 freeze-thaw cycles (−20 °C for 16 h and +4 °C for 8 h). Namely, the addition of GF resulted in 5 and 3% higher *f_fl_* of glass fiber reinforced mortars subjected to a freeze-thaw process for mixtures with 40 and 50% replacement of fine sand by marble dust, respectively in comparison to mixes without fibers. Furthermore, changing the dosage of fibers from 0.50 kg/m^3^ to 0.75 kg/m^3^ allowed to improve the flexural strength by 5% when compared with plain mortars exposed to freezing and thawing cycles. In [115], the complex study on the freeze-thaw resistance of FRC was performed. In Figure 26, the relative dynamic elastic modulus for different FRC subjected to 25–100 freeze-thaw cycles is shown. It can be stated that GFRC had the smallest losses with the increased number of cycles and its behavior was significantly improved when compared with PC. Moreover, concrete with GF outperformed the results for mixes with BF and especially with PPF. Lei et al. [46] incorporated 1.0 kg/m^3^ of GF with short (3 mm) to long (19 mm) fibers mass ratios equaled to 0:0, 0:1, 3:7, 5:5, 7:3, and 1:0 for which *f_c_* of concrete decreased by around 22, 9, 11, 7.5, 12, 16.5%, subsequently. Taking into consideration that all samples were frozen for 4 h at −20 °C and then melted for 4 h at +20 °C for 50 salt-solution freeze-thaw cycles, it was concluded that GF enhanced the resistance and durability of concrete. Additionally, within this study, the synergistic influence of PPF and GF on salt-solution freeze-thaw resistance was proven. Regarding the chloride freeze-thaw cycles, Yin et al. [116] claimed that GFRC flexural and average interfacial bonding strength was greater compared with PC. To sum up, the enhancement of concrete freeze-thaw resistance when GF were added is real and considerable. Because water, entrapped in the pores in minus temperatures, freezes and as a result expands its volume, tensile stresses occur. GFRC with its improved mechanical properties, especially tensile strength, decreases the freeze-thaw deterioration and limits the expansion of concrete and its cracking. Finally, the inclusion of fibers can prolong the service-life of the element since concrete can resist more freeze-thaw cycles.

#### 4.9.3. Corrosion Resistance

**Figure 27:**
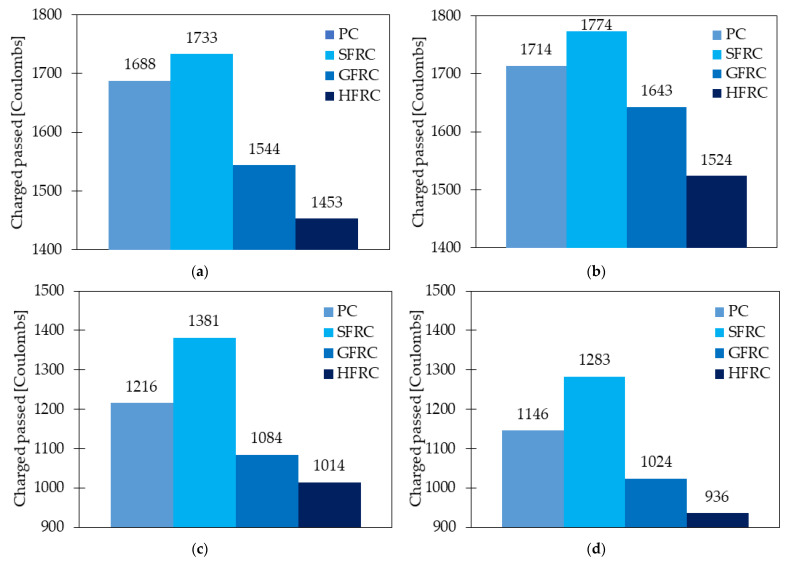
Chloride penetration test coefficients: (**a**) mix type 1—concrete grade 40 and sand to aggregate ratio 0.50; (**b**) mix type 2—concrete grade 40 and sand to aggregate ratio 0.57; (**c**) mix type 3—concrete grade 80 and sand to aggregate ratio 0.50; (**d**) mix type 4—concrete grade 80 and sand to aggregate ratio 0.57 [21].

One of the parameters that characterizes concrete corrosion durability is electrical resistance. The resistivity of concrete describes sample water saturation degree, chloride penetration resistance, or the corrosion rate. It is one of the criteria for judging the risk of corrosion, namely, higher concrete resistivity means lower corrosion danger. Moghadam et al. concluded in their research [33] that the addition of GF allowed for almost 34% improvement of the electrical resistivity. In [21], the corrosion probability at 90 days for GFRC was equaled <5%, while for concrete without fibers and with SF the probability was greater. This is attributed to the fiber bridging nature, however, taking into consideration also the fact that GF are poor conductors of electricity and SF have high conductivity and hence they behaved the worst according to this article. Ganta et al. [21] measured also the chloride diffusivity according to standard [117]. The chloride permeability is lower for smaller values of passed charges. In Figure 27, the results of the chloride penetration test are presented from which it can be concluded that HFRC with SF and GF performed best, followed by mixtures with only GF. Furthermore, the electrical resistivity of GFRC mixes was higher than that of PC mixes. Another test to research corrosion durability is a rapid chloride migration test performed in [26]. In the paper, owing to the addition of GF, PPF, and GF + PPF both the migration depth of chloride and chloride migration coefficient decreased. Additionally, by increasing the dosage of fibers, the concrete resistance to chlorides was improved continuously. Specifically, for *V_f_* = 1.5%, the chloride migration depth for GFRC, PPFRC, and HFRC was 22.7, 19.8, and 19.5 mm subsequently, while for PC around 24.1 mm. As a result, the property reduced by 10.9, 21.7, and 23.3% compared with concrete without fibers. Considering the classifications of chloride penetration resistance, FRC mixtures belonged to the high or very high class. This improved behavior of concretes with fibers was related to the ability of shrinkage crack limitation and porosity reduction. Similar conclusions were obtained in the rapid chloride penetration test. The highest influence of fibers addition was observed for *V_f_* = 1.5%. Namely, the charges passed of concretes with GF, PPF, and GF + PPF were reduced by 6.77, 11.3, and 20.15% compared with PC. This meant that the results for PC, GFRC, PPFRC, and HFRC equaled 3626, 3381, 3217, and 2896 C respectively. Ratha et al. [118] added to the concrete mixture *V_f_* = 0.1% of GF (*l_f_/d_f_* = 12 mm/14 μm). For that reason, the electrical resistivity increased 1.40 times when compared with PC which meant good resistance against corrosion. Another important study done by Atewi et al. [119] showed that the incorporation of 1.5% of GF resulted in the decrease of the total charge passed from 3068 to 3004 C. Finally, a comprehensive study on GFRC performance [27] revealed that with the addition of GF, the rebar mass loss subjected to corrosion processes decreased (Table 5). The test was performed according to ASTM G109-07 [120] which is dedicated to determining the influence of chemical admixtures on the corrosion level of steel bars embedded in concrete specimens subjected to chloride environments. The mass of the bar was measured before and after the sample treatment. The durability improvement is attributed to the reduction of crack widths and pores which limited the ingress possibility of harmful agents. To sum up, it can be said that GFRC can fulfill the requirements of a smart material, that is more durable, resistant to corrosion and other harmful agents.

### 4.10. Bonding Strength 

**Figure 28:**
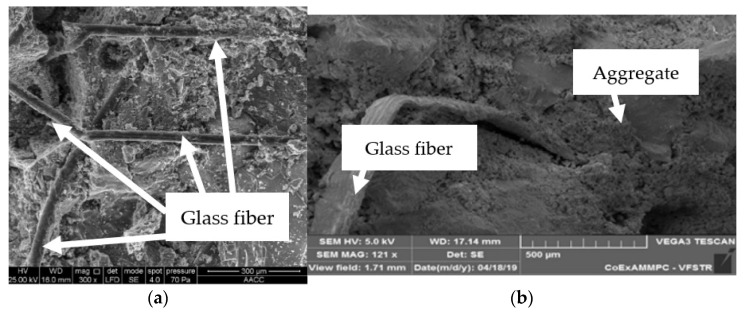
SEM image of GFRC microstructure: (**a**) [116] Adapted permission from Ref. [116]. Copyright 2019 Elsevier; (**b**) [27].

Yin et al. [116] concluded that the inclusion of GF enhanced the bonding strength between fibers and matrix subjected to the chloride freeze-thaw cycles as well as chloride wet-dry cycles when compared with ordinary concrete. The maximum pull-out force was measured and then the average bonding strength was calculated. In Figure 28a, a scanning electron microscopy (SEM) image of GFRC microstructure is seen. It can be mentioned that for the first type of treatment the bonding strength of samples with polyvinyl alcohol fibers (PVAF) was worse than that for PC samples and had a downward trend when the number of cycles increased. GFRC average interfacial bonding strength slightly improved under chloride freeze-thaw cycles. On the other hand, for the wet-dry cycles, the behavior of PVAF surpassed GF. However, the positive effect of fibers was explained by crack bridging abilities and increased tensile strength. Furthermore, in [27] it was concluded that when fibers were properly packed with the surrounding concrete matrix, a good bonding strength was achieved (Figure 28b). Moreover, the good packing might have been the reason for the limitation of cracks while shrinkage and reduction of voids sizes occurred. According to research work presented in [26], GF filled voids and decreased the concrete permeability. It was also established that a hybrid mixture with both GF and PPF gave the best results considering the size of voids. Namely, smaller in diameter PPF were able to connect smaller voids, while thicker GF, bigger ones. To sum up, owing to the addition of GF, harder to break as well as denser and more impermeable material can be produced. This conclusion is also in agreement with the findings of Chen et al. in [74]. Additionally, they revealed the positive effect of SF on the performance of glass fibers reinforced mortars since it protected fibers from aging caused by erosion of the alkali environment. Namely, SF by covering the GF surface created the barrier between fiber and alkali environment. In SEM analyses, some works indicated the tendency of GF to form bundles and flocculation which led to nonuniform fiber distribution as well as weakening of the bonding strength [35]. As a result, attention must be paid while mixing concrete and choosing optimal fiber dosage.

### 4.11. Eco-Friendly and Economic

The concept of a Smart City is strongly connected with the idea of sustainable development. As a result, the eco-friendly and economic background of GFRC should be also taken into consideration like it was done for other FRC, for instance, PPFRC in [121]. It has been emphasized before that improvements in the mechanical performance of GFRC allow creating stronger and more resistant concrete. As a result, slenderer elements may be manufactured with less material required. Additional advantage flows from the enhanced durability since the service life is extended and maintenance processes require less effort. The increased corrosion resistance has to be highlighted, along with additional benefits resulting from the absence of steel fibers and ordinary reinforcement.

The environmental and economic characteristic of GFRC was discussed in [122], where it was stated that concretes with GF are a more costly materials than PC. Namely, the cost of 1 m^3^ of GFRC with 0.5 and 1.0% fibers equaled 96 and 107.2 USD/m^3^, respectively, while the price of concrete without fibers was 81.4 USD/m^3^. However, it must be mentioned that cost of GF was almost 7 and 17% smaller than the price of SF and PPF, respectively. Nevertheless, since GF are lighter than SF but heavier than PPF, the total USD/m^3^ of GFRC exceeded the cost of PPFRC, although was smaller than the cost of SFRC. For instance, *V_f_* = 1.0% for SFRC required 78 kg/m^3^ of SF, GFRC required 26 kg/m^3^ of GF, and PPFRC required only 9 kg/m^3^ of PPF. On the other hand, when taking into consideration the enhanced mechanical properties of GFRC, it was possible to decrease the thickness of the designed concrete pavement. Due to this, the slab depth could have been around 55 and 65 mm smaller for GFRC with *V_f_* = 0.5 and 1.0% of GF than the one made from PC. It must be mentioned that the thickness reduction was comparable with the reduction for SFRC and greater than that for PPFRC. In consequence, the overall cost of GFRC pavement (USD/m^2^) was smaller than that of PC and equaled 5–10% less depending on the GF dosage. Regarding carbon emission, when comparing the production of CO_2_ kg/m^3^ the values for GFRC would have been greater than PC. They increased from 356 kg/m^3^ to 382 and 408 kg/m^3^ of CO_2_ emission, so by 7 and 15% for 0.5 and 1.0% of GF addition, respectively. Nevertheless, when analyzing the carbon footprint and CO_2_ production per m^2^ the conclusions were different. It occurred that GFRC were composites eco-friendlier by around 17% than concrete without fibers. This is in agreement with the findings of Hussain et al. [123], where it was confirmed that the addition of 1% of GF to normal and high strength concrete reduced the required thickness of concrete pavement by around 23 and 35%, respectively. Additionally, normal strength GFRC pavement had a smaller thickness than high strength PC. Similarly to [122], the overall cost USD/m^3^ was higher when compared with PC, namely, 1.31 and 1.14 times for normal and high strength GFRC. Nevertheless, while studying costs per m^2^ it appeared that normal strength PC cost 15.06 USD/m^2^ and GFRC 14.91 USD/m^2^; high strength PC cost 15.54 USD/m^2^ and GFRC 14.38 USD/m^2^.

## 5. Application of GFRC

GFRC is an increasingly popular material and is more and more widely used. Nowadays, not only structural elements are made with the use of GF but also elements accompanying the building process, as well as elements of small architecture to create more attractive, durable, and safer public spaces.

**Figure 29:**
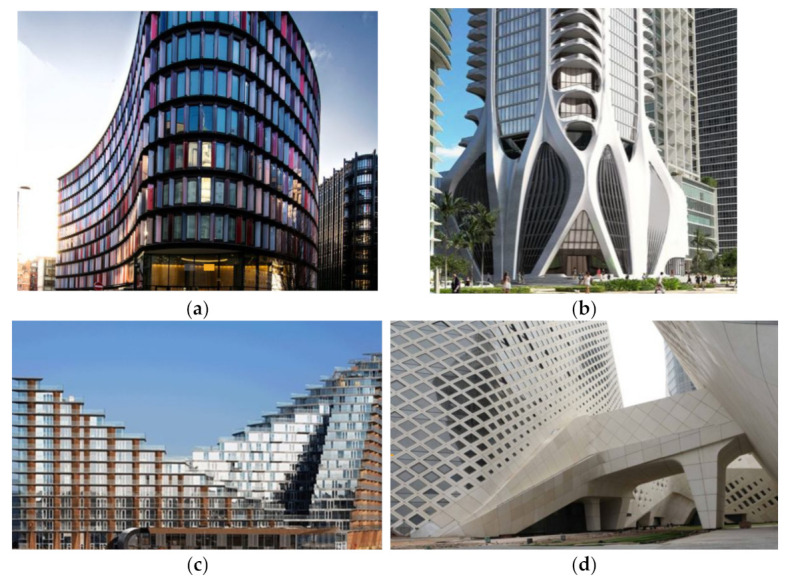
(**a**) Two New Ludgate, London, UK [124]; (**b**) 1000 Museum, Miami, USA [124]; (**c**) AARhus, Aarhus, Denmark [124]; (**d**) Nanjing Youth Olympic Conference Center, Nankin, China [124].

GFRC is used for the creation of prefabricated façades panels of residential and cultural buildings, airports, stadiums, museums, and galleries (Figure 29). They have not only higher strength than ordinary reinforced concrete façades, but also, they are characterized by enhanced resistance to temperature changes, sun, pollution, corrosion, and have hydrophobic features. That is why they are so eagerly used to construct elements of outdoor architecture. They can also be called vandal-proof and graffiti-proof [9] due to their hydrophobic properties and high impact resistance. It is crucial, especially in urban areas where there is an increased risk of vandalism. This also fulfills the requirements of public safety and intelligent buildings. Additionally, they are very popular when more design freedom is required and the building shape expects to be more complicated, modern, and unusual. GFRC elements on Two New Ludgate in London allowed for achieving a streamlined and dynamic impression of the building (Figure 29a). Similarly, the exoskeleton of the 1000 Museum in Miami was constructed using GFRC (Figure 29b). This uncommon structural solution was applied to maximize the useable area of open spaces in the building allowing for some columns elimination and wall thickness reduction. Another example can be Aarhus in Denmark, where GFRC façade claddings allowed to express the intention of the architects to shape it as two AA’s referring to the name of the city (Figure 29c). Furthermore, in the case of the Nanjing Youth Olympic Conference Center in China, the GFRC panels were covered with special inorganic hybrid silicone-acrylate emulsion providing i.e., hydrophobic properties (Figure 29d).

**Figure 30:**
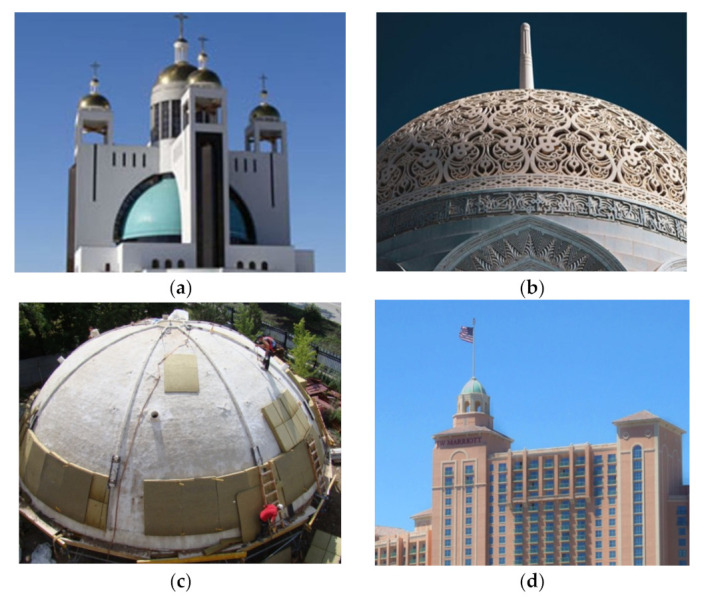
(**a**) Cathedral of the Resurrection of Jesus Christ, Kyiv, Ukraine [125]; (**b**) Mohammed Al-Ameen Mosque, Muscat, Oman [126]; (**c**) church dome, Ukraine [127]; (**d**) Marriott—Grande Lakes Resort, Orlando, USA [128].

The next field of GFRC application are domes which can occur in palaces, shopping centers, universities, theaters, stadiums, and others (Figure 30). There are several advantages of concrete with fibers over ordinary reinforced concrete in this kind of structures. First, its high mechanical properties are comparable with concrete reinforced traditionally allowing for the construction of domes when their diameter is greater than 20 m. Second, the shape of domes increases the difficulty of placing and bending the reinforcing bars in contrast to the concrete mixture with already embedded reinforcement in form of fibers. Furthermore, GFRC can be applied by spraying as shotcrete which facilitates its application. Additionally, very often in the case of domes, a high-quality concrete finish is required to enable decorative painting such as in the Cathedral of the Resurrection of Jesus Christ in Kyiv (Figure 30a). Another time, GFRC dome segments need to provide the required smoothness for attaching the designed cover i.e., ceramic tiles in one of the domes of the Presidential Palace in Abu Dhabi where the tolerance could have been not more than 2 mm. Finally, domes constructed from GFRC allow for the creation of not only plain surfaces but also patterned ones like in a dome of Mohammed Al-Ameen Mosque in Muscat (Figure 30b). To sum up, owing to the strength, plasticity, formability, lightness, and coloring possibilities of GFRC, it is acknowledged to form modern and unique designs giving architects more creative possibilities. Additionally, because of their increased durability, they fulfill the requirements of sustainable development, prolonging the service life of the element, and reducing maintenance costs.

**Figure 31:**
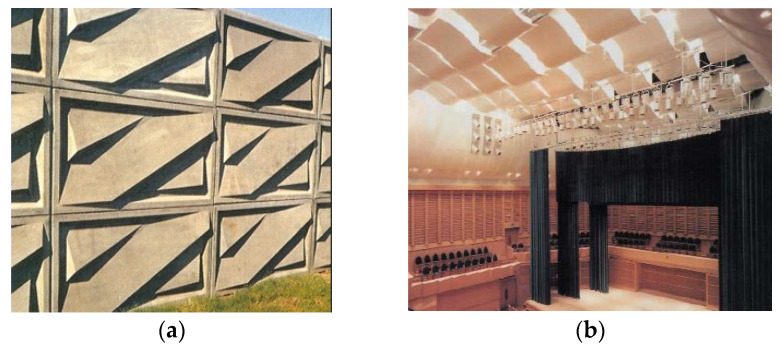
(**a**) GFRC external acoustic screens [129]; (**b**) GFRC internal acoustic panels [130].

Considering the soundproofing properties of concrete with glass fibers, GFRC acoustic panels are widely used in places when road, train, or intersection noises are bothersome (Figure 31a). The research showed that GFRC panels are more effective in noise absorption than those made from perforated aluminum. Previously mentioned plasticity of this material and freedom of shaping can increase not only the comfort of living but also the attractiveness of neighboring areas. Modern and visually pleasant GFRC sound barriers can be both functional and decorative elements of the city that can fill in the landscape [129]. However, they are not used only outside because GFRC are also very popular in creating rooms where acoustics play an important role like cinemas, theaters, concert halls, and others (Figure 31b). For example, in Kilden Performing Arts Centre on Odderøya in Kristiansand in Norway, the internal acoustic panels were used because of their soundproofing features, lightweight allowing for lifting them by available on-site cantilever crane devices, easy fixing solution leading to speed up the installation process, and supreme finish quality possible to achieve even for the complex geometry of GFRC panels.

**Figure 32:**
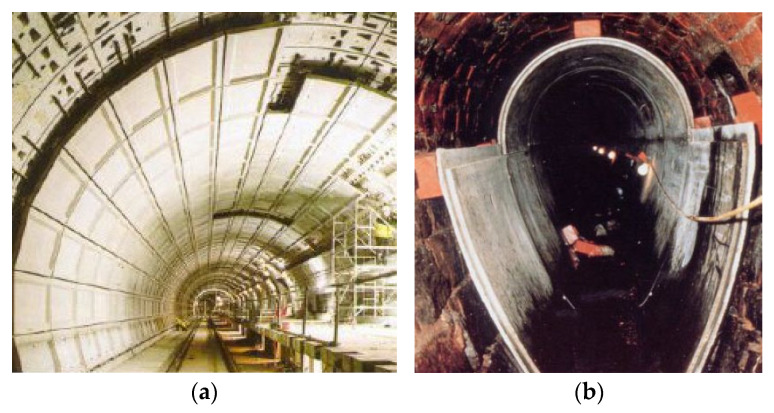

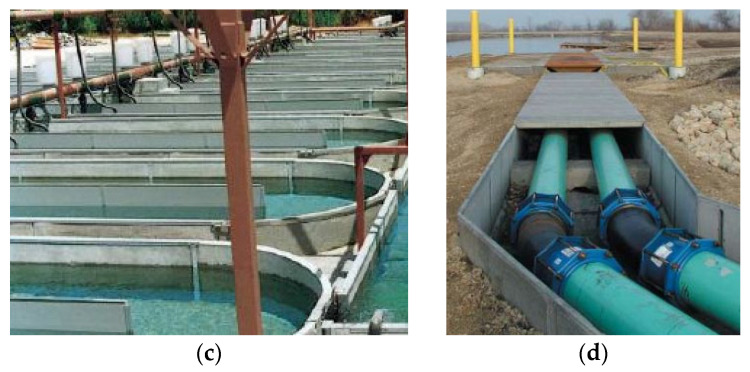
(**a**) GFRC tunnel panels [131]; (**b**) GFRC municipal sewerage panels [132]; (**c**) GFRC melioration system [133]; (**d**) GFRC ducts [134].

**Figure 33:**
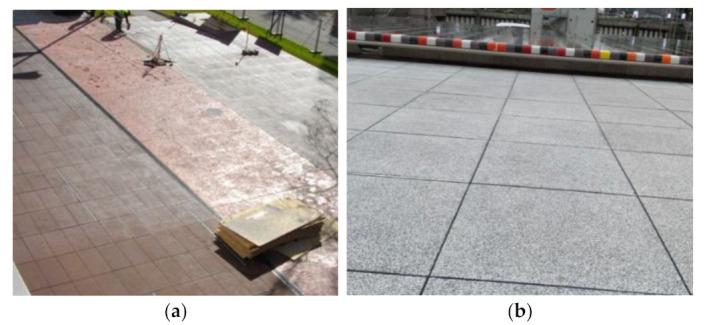
(**a**) GFRC pavement [135]; (**b**) GFRC pavement on La Défense, Paris, France [136].

Furthermore, GFRC are also an incredibly valued material in infrastructure. Primarily due to their high strength, durability, low weight, ease, and speed in handling and assembling. Second, advantages deriving from its fire, corrosion resistance, and other high tolerances on unfavorable environmental conditions make GFRC a suitable material for bridges, viaducts, tunnels, retaining walls, and protective channels for electric cables and pipes (Figure 32). Additionally, successful crack limitation owing to the inclusion of GF allows GFRC to be used in the production of any elements requiring water and frost-proof resistance such as melioration and drainage systems, ducts, as well as in municipal sewage systems (Figure 32). For example, in England, Holland, and France, sewers made from bricks were lined with GFRC formworks and then grouted. This solution has also the additional advantage of providing a smooth surface to enable better hydraulic performance. Because pavements are subjected to abrasion, impact damages, road salts, exhaust gases, dust, and weather changes during daily life, GFRC as a materials of good abrasion, impact, freeze-thaw, fire, and chemical resistance are ideal to produce road and paving slabs (Figure 33). Moreover, GFRC slabs are readily used to create pavements and other elements in marine environments since they have increased durability and corrosion resistance.

**Figure 34:**
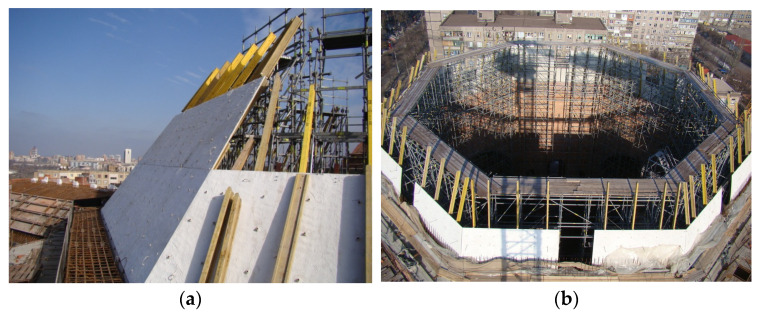
GFRC permanent formwork: (**a**) Church in Donetsk, Ukraine [137]; (**b**) Church in Mariupol, Ukraine [138].

Concrete with GF is also used to create permanent formworks (Figure 34). These are formworks that stay in place and act as an additional stabilizer, reinforcement, and protection: water, fire, corrosion, chemical, and against unfavorable weather conditions. For example, they are very popular in the concreting process of previously mentioned water structures, tunnels, silos chutes, as well as domes, and arched floors. Moreover, very often they are applied in projects where the places are hard to reach. Additionally, owing to them the construction time is faster, easier, and the erection process safer for workers.

**Figure 35:**
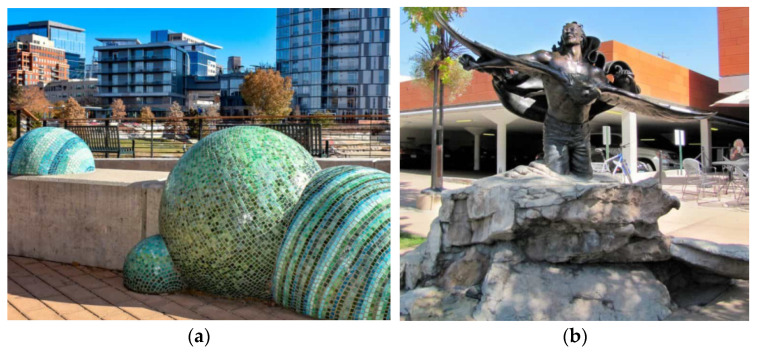
(**a**) GFRC “Sing and Glide” sculpture in Confluence Park in Denver, USA [139]; (**b**) GFRC base of “I Too Know The Eagle” sculpture in Cherry Creek North in Denver, USA [139].

**Figure 36:**
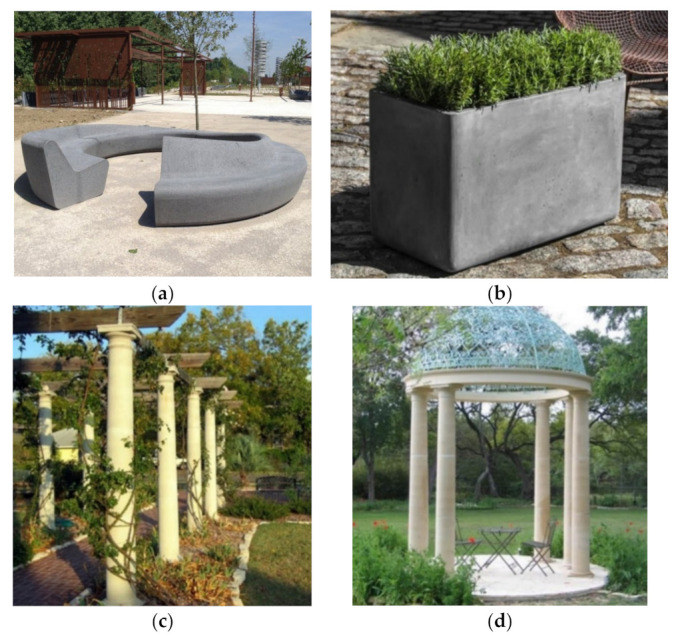
(**a**) GFRC bench [140]; (**b**) GFRC flower pot [141]; (**c**) GFRC pergola [142]; (**d**) GFRC gazebo [143].

It is noteworthy that owing to the high plasticity, high strength, and low weight of GFRC, decreased thickness as well as freedom of shaping an original and unique elements can be created, both large and small. Namely, GFRC applies also to small aesthetic elements like sculptures (Figure 35), cornices, entryways, bas-reliefs, window surrounds, capitols, and functional components, such as tables, benches, fences, and flower pots (Figure 36). For example, in Confluence Park in Denver, the sculpture in a form of spheres called “Sing and Glide“ designed by Jeanne Quinn and covered in mosaic tiles is made from concrete with GF (Figure 35a). Similarly, the base of the sculpture “I Too Know The Eagle” in Cherry Creek North in Denver is made from GFRC (Figure 35b). Repeatedly, GFRC are used to cast columns of pergolas or gazebos. It must be mentioned that GFRC can be colored with oxides which increases its attractiveness for creating elements of public use.

**Figure 37:**
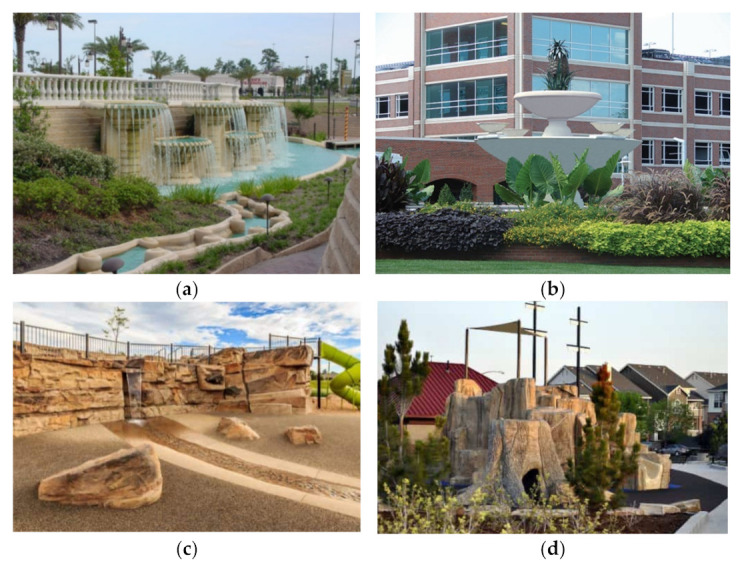
(**a**,**b**) GFRC fountain [144,145]; (**c**) Mehaffey Park in Loveland in Colorado, USA [146]; (**d**) Center Park in Westminster in Colorado, USA [147].

Nowadays, one of the main streams of GFRC application is landscaping and the creation of natural-looking waterfalls, fountains, rocks, and cliffs (Figure 37). This idea was used in the construction of a waterfall in a park in Owasso, a fountain on the Streets at South Glenn, rockwork in Patricia and J. Spencer Standish Garden, and Johnson Habitat Park; moreover, in Mehaffey Park to refer to the nature of the Colorado landscape, the rock wall, landing pad from the bridge, small rocks on the ground, and fallen logs were manufactured from GFRC (Figure 37c). Such play spaces allow children to develop not only their motor skills by climbing, gripping but also balance and coordination. The playground in Center Park in Westminster has the shape of a pirate boat where the slide, holes, and logs are built with the addition of GF (Figure 37d).

The places of the GFRC application mentioned so far were related to the construction of new elements. However, it must be mentioned that concrete with GF is also used for the reconstruction of damaged elements of monuments and architectural details. Considering wide possibilities in shape formation and coloring to reproduce the original look, it is a suitable material for this purpose.

## 6. Conclusions and Future Perspective

In Smart City 3.0, more and more emphasis is placed on smart manufacturing, smart buildings, and smart environment. Due to this, the idea of sustainable development is one of the most important current global concerns. The concrete industry, as responsible for 8–9% of total C0_2_ in the atmosphere, has to face this challenge and adjust to manufacturing more durable building materials, slenderer concrete elements with extended service life. GFRC can meet these requirements better than ordinary concrete. As a result, it is essential to research both its reasonable structural use, as well as its physical and mechanical advantages. 

In Table 6 the influence of GF inclusion on different concrete properties when compared with plain concrete is presented. The mechanical advantages of GFRC over concrete without fibers arise from the fact that tensile strength is enhanced usually up to 50% which allows producing smaller cross-sections of elements. Additionally, a decrease in crack widths up to 27 times and around 50% reduction in the total crack area makes GFRC elements better protected from ingress of harmful agents and action of unfavorable environmental conditions than ordinary concrete. Moreover, decreased thermal conductivity allows for better energy performance from the building’s point of view. This results in cheaper structures both in manufacturing and maintaining even though GFRC are more expensive materials. On the other hand, limiting brittle failure by improved toughness, as well as higher abrasion, durability, fire, and impact resistance makes Smart Cities safer and more vandal-proof places. However, it must be mentioned that the mechanical properties will be enhanced as long as sufficient workability and uniform fiber distribution are assured. Otherwise, physical and mechanical features will deteriorate as a result of bundles creation, increased porosity, and weak bonding between fibers and cementitious matrix. From the environmental point of view, GFRC are eco-friendlier materials than ordinary concretes since their application can decrease the emission of CO_2_ by 17%. The article also emphasizes on the possibility of creating not only GFRC structural elements mainly intended for load transferring but also elements accompanying the building process, as well as elements of small architecture that make public spaces more attractive, durable, and safer. It is an interesting point of view since until now only its structural nature was highlighted. Owing to greater design and shaping freedom, GFRC can also better fulfill the needs of the habitants of Smart City. GFRC may be used to manufacture:

- Pavements;
- Façades panels of residential and cultural buildings, airports, stadiums, museums, galleries, and others;
- Domes of palaces, shopping centers, universities, theaters, stadiums, and others;
- External and internal acoustic panels;
- Panels of tunnels, bridges, viaducts, and retaining walls;
- Protective channels for electric cables and pipes;
- Elements of melioration and drainage systems, ducts, and municipal sewage systems;
- Permanent formworks;
- Sculptures, cornices, entryways, bas-reliefs, window surrounds, capitols;
- Functional components such as tables, benches, fences, flower pots, gazebos, and pergolas;
- Waterfalls, fountains, rocks, and cliffs in the process of landscaping and creation of natural-looking elements;
- Repairing works, reconstructions.

## Figures and Tables

**Table 2:** Keyword methodology.

Main Keywords	Secondary Keywords	Third Keywords		
Concrete Glass fibers Glass fiber reinforced concrete	Composition Physical properties Mechanical properties Tensile strength Durability Sustainable development Application	Workability Crack limitation Shrinkage Modulus of elasticity Compressive strength Uniaxial tensile strength Flexural tensile strength Splitting tensile strength Impact resistance	Spalling resistance Fire resistance Abrasion resistance Thermal conductivity Corrosion resistance Electrical resistance Chloride resistance Freeze-thaw resistance Bonding strength	Ductility Toughness Permeability Water absorptionPorosity Eco-friendly Economic

**Table 3:** Material composition of GFRC.

Ref.	w/b	Water	SP	Cement		SF	NS	FLA	BFS	MK	Aggregates			Fibers	
											FA	CA	*D_max_*	*l_f_/d_f_*	*V_f_*
	[–]	[kg/m^3^]	[kg/m^3^]	Type	[kg/m^3^]	[kg/m^3^]	[kg/m^3^]	[kg/m^3^]	[kg/m^3^]	[kg/m^3^]	[kg/m^3^]	[kg/m^3^]	[mm]	[mm/μm]	[%]
[14]	0.50	185.0	✓	OPC 42.5	370.0						758.0	1047.0	20	6/30; 12/30	0.50;1.00;1.50
[15]	0.45	165.0	N.I.	N.I.	185.0			74.0	111.0		860.0	873.0	10	19/14	1.50
[16]	0.40	152.0	1.9	OPC 53	378.1						816.6	1104.7	20	12/14	0.50;1.00;1.50 ^(1)^
[17]	0.43	172.0	✓	OPC 53	300.0			100.0			764.0	1145.0	20	3/14; 6/14; 12/14; 20/14	0.10;0.20;0.30; 040;0.50
[18]	0.25	82.0	3.3	OPC 43	308.8			16.3			789.0	478.0	10	6/N.I.	0.50;1.00 ^(2)^
	0.25	82.0	3.3	OPC 43	292.5			32.5			789.0	478.0	10	6/N.I.	0.50;1.00 ^(2)^
	0.25	82.0	3.3	OPC 43	276.3			48.8			789.0	478.0	10	6/N.I.	0.50;1.00 ^(2)^
	0.25	82.0	3.3	OPC 43	260.0			65.0			789.0	478.0	10	6/N.I.	0.50;1.00 ^(2)^
	0.25	82.0	3.3	OPC 43	243.8			81.3			789.0	478.0	10	6/N.I.	0.50;1.00 ^(2)^
[19]	0.45	186.0	N.I.	OPC 43	330.4			82.6			706.0	1117.0	20	12/N.I.	1.00;1.50;2.00 ^(1)^
	0.45	186.0	N.I.	OPC 43	289.1			123.9			706.0	1117.0	20	12/N.I.	1.00;1.50;2.00 ^(1)^
[20]	0.50	192.0	N.I.	OPC 43	383.0						672.0	1100.0	20	12/N.I.	0.25 ^(1)^
[21]	0.51	220.9	8.4	OPC 53	290.2			144.5			786.2	840.0	20	12/14	0.05
	0.51	215.9	8.2	OPC 53	290.2			133.8			912.3	735.3	20	12/14	0.05
	0.26	167.4	9.1	OPC 53	450.0	36.6		159.7			786.2	840.0	20	12/14	0.05
	0.26	162.5	8.9	OPC 53	450.0	36.0		149.1			912.3	735.3	20	12/14	0.05
[22]	0.45	180.0	4.0	CEM I 42.5R	360.0			40.0			945.0	931.0	11.2	12/13	0.25
	0.45	180.0	4.8	CEM I 42.5R	360.0			40.0			945.0	931.0	11.2	12/13	0.50
	0.45	180.0	6.8	CEM I 42.5R	360.0			40.0			945.0	931.0	11.2	12/13	0.75
	0.45	180.0	8.0	CEM I 42.5R	360.0			40.0			945.0	931.0	11.2	12/13	1.00
[23]	0.30	129.0	6.5	OPC 42.5	280.0	22.0		86.0	43.0		655.0	1165.0	20	12/15	0.45;0.90;1.35
	0.35	129.0	5.6	OPC 42.5	240.0	18.0		74.0	37.0		677.0	1204.0	20	12/15	0.45;0.90;1.35
[24]	0.45	184.5	N.I.	PSC	410.0						688.0	1081.0	40	N.I.	0.05
	0.45	176.3	N.I.	PSC	391.8						716.0	1125.0	40	N.I.	0.03
	0.50	176.3	N.I.	PSC	352.6						585.0	1112.0	40	N.I.	0.05
	0.50	180.0	N.I.	PSC	360.0						664.0	1135.0	40	N.I.	0.03
[25]	0.40	140.0	5.0	PPC 43	350.0						873.0	1110.0	20	12/14	0.33;0.67;1.00
[26]	0.42	126.0	0.6	OPC 42.5	300.0						825.0	1100.0	20	12/40	0.50;1.00;1.50
[27]	0.45	197.0	N.I.	OPC 53	438.0						674.0	1070.0	20	12/14	1.00;1.50;2.00;2.50;3.00 ^(1)^
[28]	0.50	206.0	N.I.	OPC 43	412.0						590.0	1123.5	20	25/15	1.00;2.00;3.00
[29]	0.50	218.0	N.I.	OPC	436.0						837.3	718.5	19	6/14.2	0.50 ^(1)^
	0.50	218.0	N.I.	OPC	436.0						836.1	717.3	19	6/14.2	1.00 ^(1)^
	0.50	218.0	N.I.	OPC	436.0						834.9	716.1	19	6/14.2	1.50 ^(1)^
	0.50	218.0	N.I.	OPC	436.0						837.3	718.5	19	25.4/14.2	0.50 ^(1)^
	0.50	218.0	N.I.	OPC	436.0						836.1	717.3	19	25.4/14.2	1.00 ^(1)^
	0.50	218.0	N.I.	OPC	436.0						834.9	716.1	19	25.4/14.2	1.50 ^(1)^
[30]	0.55	195.0	0.9	OPC 43	355.0						825.0	980.0	12.5	6+12+18/14	0.25
	0.55	195.0	1.5	OPC 43	355.0						825.0	980.0	12.5	6+12+18/14	0.50
	0.55	195.0	2.3	OPC 43	355.0						825.0	980.0	12.5	6+12+18/14	0.75
	0.55	195.0	2.8	OPC 43	355.0						825.0	980.0	12.5	6+12+18/14	1.00
[31]	0.31	198.0	✓	OPC 53	500.0	38.0		100.0			664.0	970.0	10	6/13.5; 12/13.5	0.10;0.20; 0.30;0.40
[32]	0.35	324.7	9.3	N.I.	927.6						927.6	0.0	N.I.	25/16	1.50 ^(3)^
	0.35	322.0	9.2	N.I.	920.0						920.0	0.0	N.I.	25/16	2.50 ^(3)^
	0.38	321.1	8.3	N.I.	834.1	10.0					917.5	0.0	N.I.	25/16	1.50 ^(3)^
	0.40	319.6	7.9	N.I.	794.1	15.0					913.2	0.0	N.I.	25/16	1.50 ^(3)^
	0.38	320.5	8.3	N.I.	832.4					10.0	915.7	0.0	N.I.	25/16	1.50 ^(3)^
	0.40	317.9	7.9	N.I.	789.7					15.0	908.2	0.0	N.I.	25/16	1.50 ^(3)^
	0.35	324.4	9.2	N.I.	919.9		0.8				926.8	0.0	N.I.	25/16	1.50 ^(3)^
	0.35	324.1	9.1	N.I.	912.3		1.5				926.0	0.0	N.I.	25/16	1.50 ^(3)^
	0.38	318.5	8.3	N.I.	827.3	10.0					910.0	0.0	N.I.	25/16	2.50 ^(3)^
	0.39	317.0	7.9	N.I.	787.6	15.0					905.7	0.0	N.I.	25/16	2.50 ^(3)^
	0.38	317.9	8.3	N.I.	825.6					10.0	908.2	0.0	N.I.	25/16	2.50 ^(3)^
	0.39	315.3	7.8	N.I.	783.2					15.0	900.7	0.0	N.I.	25/16	2.50 ^(3)^
	0.35	321.7	9.1	N.I.	912.3		0.8				919.2	0.0	N.I.	25/16	2.50 ^(3)^
	0.35	321.4	9.0	N.I.	904.8		1.5				918.4	0.0	N.I.	25/16	2.50 ^(3)^
	0.35	318.0	9.1	N.I.	908.6						908.6	0.0	N.I.	25/16	4.04 ^(3)^
	0.35	312.7	8.9	N.I.	893.4						893.4	0.0	N.I.	25/16	5.72 ^(3)^
	0.38	314.5	8.2	N.I.	817.0	10.0					898.7	0.0	N.I.	25/16	4.40 ^(3)^
	0.39	313.1	7.8	N.I.	777.8	15.0					894.5	0.0	N.I.	25/16	4.12 ^(3)^
	0.38	313.9	8.2	N.I.	815.4					10.0	896.9	0.0	N.I.	25/16	4.29 ^(3)^
	0.39	311.3	7.7	N.I.	773.5					15.0	889.5	0.0	N.I.	25/16	4.50 ^(3)^
	0.35	317.7	9.0	N.I.	901.0		0.8				907.8	0.0	N.I.	25/16	4.50 ^(3)^
[32]	0.35	317.4	8.9	N.I.	893.6		1.5				907.0	0.0	N.I.	25/16	4.60 ^(3)^
	0.38	309.3	8.0	N.I.	803.3	10.0					883.6	0.0	N.I.	25/16	5.80 ^(3)^
	0.39	307.8	7.6	N.I.	764.8	15.0					879.5	0.0	N.I.	25/16	5.60 ^(3)^
	0.38	308.6	8.0	N.I.	801.7					10.0	881.8	0.0	N.I.	25/16	5.90 ^(3)^
	0.39	306.1	7.6	N.I.	760.5					15.0	874.6	0.0	N.I.	25/16	6.00 ^(3)^
	0.35	312.4	8.9	N.I.	885.9		0.8				892.6	0.0	N.I.	25/16	6.00 ^(3)^
	0.35	312.1	8.8	N.I.	878.6		1.5				891.8	0.0	N.I.	25/16	6.10 ^(3)^
[33]	0.50	200.0	N.I.	OPC	400.0						765.0	935.0	19	12/N.I.	0.25
[34]	0.45	157.5	N.I.	CEM I 42.5N	210.0			140.0			540.0	1107.0	25	6+18/13	0.70 ^(1)^
[35]	0.12	141.0	77.6	CEM I 42.5 R	998.8	176.3					991.1	0.0	2.5	13/18	0.50
	0.12	141.0	78.7	CEM I 42.5 R	998.8	176.3					975.0	0.0	2.5	13/18	1.00
	0.12	141.0	81.7	CEM I 42.5 R	998.8	176.3					954.5	0.0	2.5	13/18	1.50
	0.12	141.0	82.8	CEM I 42.5 R	998.8	176.3					942.7	0.0	2.5	13/18	2.00
	0.12	141.0	84.6	CEM I 42.5 R	998.8	176.3					920.8	0.0	2.5	13/18	2.50
	0.12	141.0	88.2	CEM I 42.5 R	998.8	176.3					898.7	0.0	2.5	13/18	3.00
	0.14	164.5	49.4	CEM I 42.5 R	998.8	176.3					998.0	0.0	2.5	13/18	0.50
	0.14	164.5	50.5	CEM I 42.5 R	998.8	176.3					981.9	0.0	2.5	13/18	1.00
	0.14	164.5	52.9	CEM I 42.5 R	998.8	176.3					962.9	0.0	2.5	13/18	1.50
	0.14	164.5	54.1	CEM I 42.5 R	998.8	176.3					946.8	0.0	2.5	13/18	2.00
	0.14	164.5	55.2	CEM I 42.5 R	998.8	176.3					930.6	0.0	2.5	13/18	2.50
	0.14	164.5	62.3	CEM I 42.5 R	998.8	176.3					900.1	0.0	2.5	13/18	3.00
[36]	0.45	200.6	0.0	OPC 53	450.0						751.3	1010.4	16	6/13	0.02;0.03;0.04
	0.38	211.7	4.5	OPC 53	430.0			133.2			853.1	782.6	16	6/13	0.02;0.03;0.04
[37]	0.45	186.7	N.I.	PC	415.0						701.4	1060.0	N.I.	21.7/N.I.; 17.2/N.I.; 21.2/N.I.; 19.5/N.I.	1.20
	0.45	186.7	N.I.	PC	415.0						701.6	1060.2	N.I.	25.4/N.I.	1.20
[38]	0.55	187.6	N.I.	N.I.	306.9			34.1			751.0	988.0	20	12/N.I.	0.19
	0.40	350.4	N.I.	N.I.	788.4			87.6			578.0	760.0	20	12/N.I.	0.37
	0.40	350.4	N.I.	N.I.	788.4			87.6			578.0	760.0	20	12/N.I.	0.74
	0.55	187.6	N.I.	N.I.	306.9			34.1			751.0	988.0	20	40/N.I.	0.19
	0.40	350.4	N.I.	N.I.	788.4			87.6			578.0	760.0	20	12/N.I.	0.02
	0.40	350.4	N.I.	N.I.	788.4			87.6			578.0	760.0	20	24/N.I.	0.02

N.I.—no information; ^(1)^—% of cement weight; ^(2)^—% of concrete volume; ^(3)^—% of concrete weight.

**Table 4:** Physical and mechanical characterization of PC and GFRC.

Ref.	Characterization of Fibers				Slump		V-Funnel		*E_cm_*		*f_c_*		*f_spl_*		*f_t_*		*f_fl_*	
	*l_f_/d_f_*	Material	*V_f_*	*f_ft_*														
	[mm/μm]		[%]	[MPa]	[mm]		[s]		[GPa]		[MPa]		[MPa]		[MPa]		[MPa]	
[14]	-	-	0.00	-											2.58	100%		
	6/30	AR-GF	0.50	1700											2.69	104%		
	6/30	AR-GF	1.00	1700											2.73	106%		
	6/30	AR-GF	1.50	1700											2.61	101%		
	12/30	AR-GF	0.50	1700											2.73	106%		
	12/30	AR-GF	1.00	1700											2.98	116%		
	12/30	AR-GF	1.50	1700											2.79	108%		
[15]	-	-	0.00	-							46.0	100%						
	19/14	GF	1.50								37.0	80%						
	19/55	PPF	0.15								42.0	91%						
[16]	-	-	0.00 ^(1)^	-	62	100%					51.0	100%						
	12/14	GF	0.50 ^(1)^		48	77%					53.5	105%						
	12/14	GF	1.00 ^(1)^		37	60%					52.0	102%						
	12/14	GF	1.50 ^(1)^		25	40%					51.5	101%						
[17]	-	-	0.00	-	160	100%									3.68	100%		
	3/14	GF	0.10	1700	126	79%									3.95	107%		
	6/14	GF	0.10	1700	119	74%									3.86	105%		
	12/14	GF	0.10	1700	110	69%									3.78	103%		
	20/14	GF	0.10	1700	106	66%									3.71	101%		
	3/14	GF	0.20	1700	112	70%									4.07	111%		
	6/14	GF	0.20	1700	109	68%									3.99	108%		
	12/14	GF	0.20	1700	101	63%									3.87	105%		
	20/14	GF	0.20	1700	98	61%									3.77	102%		
	3/14	GF	0.30	1700	105	66%									4.37	119%		
	6/14	GF	0.30	1700	103	64%									4.21	114%		
	12/14	GF	0.30	1700	94	59%									4.08	111%		
	20/14	GF	0.30	1700	88	55%									3.93	107%		
	3/14	GF	0.40	1700	67	42%									4.02	109%		
	6/14	GF	0.40	1700	64	40%									3.90	106%		
	12/14	GF	0.40	1700	56	35%									3.78	103%		
	20/14	GF	0.40	1700	52	33%									3.75	102%		
[17]	3/14	GF	0.50	1700	50	31%									3.93	107%		
	6/14	GF	0.50	1700	46	29%									3.85	105%		
	12/14	GF	0.50	1700	43	27%									3.76	102%		
	20/14	GF	0.50	1700	39	24%									3.69	100%		
	3 + 6/14	GF	0.30 (0.06 + 0.24)	1700	92	58%									4.30	117%		
	3 + 6/14	GF	0.30 (0.12 + 0.18)	1700	126	79%									5.19	141%		
	3 + 6/14	GF	0.30 (0.15 + 0.15)	1700	116	73%									5.06	138%		
	3 + 6/14	GF	0.30 (0.18 + 0.12)	1700	109	68%									4.68	127%		
	3 + 6/14	GF	0.30 (0.24 + 0.06)	1700	90	56%									4.30	117%		
	12 + 20/14	GF	0.30 (0.06 + 0.24)	1700	81	51%									3.75	102%		
	12 + 20/14	GF	0.30 (0.12 + 0.18)	1700	112	70%									4.43	120%		
	12 + 20/14	GF	0.30 (0.15 + 0.15)	1700	103	64%									4.30	117%		
	12 + 20/14	GF	0.30 (0.18 + 0.12)	1700	97	61%									4.05	110%		
	12 + 20/14	GF	0.30 (0.24 + 0.06)	1700	79	49%									3.80	103%		
	3 + 6+12 + 20/14	GF	0.30 (0.02 + 0.04 + 0.10 + 0.14)	1700	108	68%									4.52	123%		
	3 + 6+12 + 20/14	GF	0.30 (0.05 + 0.07 + 0.07 + 0.11)	1700	140	88%									5.50	149%		
	3 + 6+12 + 20/14	GF	0.30 (0.06 + 0.09 + 0.06 + 0.09)	1700	129	81%									5.38	146%		
	3 + 6+12 + 20/14	GF	0.30 (0.07 + 0.11 + 0.05 + 0.07)	1700	121	76%									4.89	133%		
	3 + 6+12 + 20/14	GF	0.30 (0.10 + 0.14 + 0.02 + 0.04)	1700	106	66%									4.55	124%		
[19]	- ^(I)^	-	0.00 ^(1)^	-							16.6	100%						
	12/N.I. ^(I)^	GF	1.00 ^(1)^								23.8	144%						
	12/N.I. ^(I)^	GF	1.50 ^(1)^								24.7	149%						
	12/N.I. ^(I)^	GF	2.00 ^(1)^								26.0	157%						
	- ^(II)^	-	0.00 ^(1)^	-							16.3	100%						
	12/N.I. ^(II)^	GF	1.00 ^(1)^								20.5	126%						
	12/N.I. ^(II)^	GF	1.50 ^(1)^								21.3	131%						
	12/N.I. ^(II)^	GF	2.00 ^(1)^								22.7	140%						
	50/1000 ^(I)^	SF	1.00 ^(1)^								18.7	113%						
	50/1000 ^(I)^	SF	1.50 ^(1)^								20.5	124%						
	50/1000 ^(I)^	SF	2.00 ^(1)^								23.4	141%						
	50/1000 ^(II)^	SF	1.00 ^(1)^								19.3	119%						
	50/1000 ^(II)^	SF	1.50 ^(1)^								20.3	125%						
	50/1000 ^(II)^	SF	2.00 ^(1)^								21.6	133%						
[21]	- ^(I)^	-	0.00	-	730 ^(2)^	100%	7.12	100%			49.5	100%	4.10	100%			5.15	100%
	- ^(II)^	-	0.00	-	750 ^(2)^	100%	7.81	100%			51.2	100%	4.59	100%			5.48	100%
	- ^(III)^	-	0.00	-	742 ^(2)^	100%	6.41	100%			91.1	100%	6.51	100%			6.89	100%
[21]	- ^(IV)^	-	0.00	-	725 ^(2)^	100%	7.03	100%			93.0	100%	6.39	100%			6.93	100%
	12/500 ^(I)^	SF	1.00		668 ^(2)^	92%	10.00	141%			55.5	112%	4.59	112%			5.67	110%
	12/14 ^(I)^	GF	0.05		712 ^(2)^	98%	9.23	130%			52.3	106%	4.47	109%			6.70	130%
	12/500 + 12/14 ^(I)^	SF + GF	1.50 (1.0 + 0.05)		706 ^(2)^	97%	9.62	135%			57.0	115%	4.55	111%			6.85	133%
	12/500 ^(II)^	SF	1.00		680 ^(2)^	91%	9.12	117%			56.6	111%	5.14	112%			6.03	110%
	12/14 ^(II)^	GF	0.05		740 ^(2)^	99%	8.56	110%			53.5	104%	5.00	109%			7.12	130%
	12/500 + 12/14 ^(II)^	SF + GF	1.50 (1.0 + 0.05)		725 ^(2)^	97%	8.73	112%			59.4	116%	5.09	111%			7.29	133%
	12/500 ^(III)^	SF	1.00		675 ^(2)^	91%	10.30	161%			99.4	109%	6.62	102%			7.17	104%
	12/14 ^(III)^	GF	0.05		700 ^(2)^	94%	8.35	130%			95.2	105%	6.53	100%			7.58	110%
	12/500 + 12/14 ^(III)^	SF + GF	1.50 (1.0 + 0.05)		695 ^(2)^	94%	9.04	141%			101.0	111%	6.71	103%			7.79	113%
	12/500 ^(IV)^	SF	1.00		650 ^(2)^	90%	10.50	150%			100.4	108%	6.64	104%			7.21	104%
	12/14 ^(IV)^	GF	0.05		710 ^(2)^	98%	8.65	123%			96.1	103%	6.44	101%			7.62	110%
	12/500 + 12/14 ^(IV)^	SF + GF	1.50 (1.0 + 0.05)		685 ^(2)^	94%	9.34	133%			103.1	111%	6.72	105%			7.83	113%
[22]	-	-	0.00	-	180	100%												
	12/13	GF	0.25	3400	120	67%												
	12/13	GF	0.50	3400	80	44%												
	12/13	GF	0.75	3400	110	61%												
	12/13	GF	1.00	3400	80	44%												
[23]	- ^(I)^	-	0.00	-							28.8	100%					5.46	100%
	12/15 ^(I)^	GF	0.45	1300							35.5	123%					4.75	87%
	12/15 ^(I)^	GF	0.90	1300							42.2	147%					6.26	115%
	12/15 ^(I)^	GF	1.35	1300							43.9	152%					7.00	128%
	12/60 ^(I)^	PPF	0.45	486							24.4	85%					4.68	86%
	12/60 ^(I)^	PPF	0.90	486							24.8	86%					4.64	85%
	12/60 ^(I)^	PPF	1.35	486							18.0	63%					4.16	76%
	- ^(II)^	-	0.00	-							30.2	100%					4.31	100%
	12/15 ^(II)^	GF	0.45	1300							37.9	125%					5.30	123%
	12/15 ^(II)^	GF	0.90	1300							42.6	141%					6.41	149%
	12/15 ^(II)^	GF	1.35	1300							22.3	74%					5.35	124%
	12/60 ^(II)^	PPF	0.45	486							27.9	92%					4.71	109%
	12/60 ^(II)^	PPF	0.90	486							22.4	74%					4.10	95%
	12/60 ^(II)^	PPF	1.35	486							8.5	28%					2.22	52%
[24]	- ^(I)^	-	0.00	-	30	100%					38.1	100%					2.90	100%
	N.I. ^(I)^	AR-GF	0.05		20	67%					37.0	97%					2.90	100%
	N.I. ^(I)^	AR-GF	0.03		25	83%					40.2	106%					3.10	107%
[24]	- ^(II)^	-	0.00	-	50	100%					31.0	100%					2.60	100%
	N.I. ^(II)^	AR-GF	0.05		30	60%					31.9	103%					2.70	104%
	N.I. ^(II)^	AR-GF	0.03		40	80%					33.0	106%					2.80	108%
[25]	-	-	0.00	-							30.0	100%					3.19	100%
	12/14	AR-GF	0.33	1700							41.0	137%					7.31	229%
	12/14	AR-GF	0.67	1700							30.0	100%					7.59	238%
	12/14	AR-GF	1.00	1700							28.7	96%					7.07	222%
[26]	-	-	0.00	-							30.5	100%					6.09	100%
	12/40	GF	0.50	1200							30.6	100%					6.26	103%
	12/15	PPF	0.50	276							30.7	101%					6.46	106%
	12/40 + 12/15	GF + PPF	0.50 (0.25 + 0.25)	1200 + 276							32.8	108%					6.56	108%
	12/40	GF	1.00	1200							30.9	101%					6.57	108%
	12/15	PPF	1.00	276							31.2	102%					6.62	109%
	12/40 + 12/15	GF + PPF	1.00 (0.50 + 0.50)	1200 + 276							33.6	110%					7.25	119%
	12/40	GF	1.50	1200							31.8	104%					7.10	117%
	12/15	PPF	1.50	276							32.3	106%					7.18	118%
	12/40 + 12/15	GF + PPF	1.50 (0.75 + 0.75)	1200 + 276							33.3	109%					7.33	120%
[27]	-	-	0.00 ^(1)^	-	100	100%					52.2	100%	4.56	100%			4.72	100%
	12/14	AR-GF	1.00 ^(1)^								56.0	107%	5.55	122%			5.61	119%
	12/14	AR-GF	1.50 ^(1)^								58.8	113%	5.76	126%			5.81	123%
	12/14	AR-GF	2.00 ^(1)^								62.0	119%	6.32	139%			6.45	137%
	12/14	AR-GF	2.50 ^(1)^								60.7	116%	6.42	141%			5.74	122%
	12/14	AR-GF	3.00 ^(1)^		74	74%					54.5	105%	4.94	108%			5.61	119%
[28]	-	-	0.00	-							21.2	100%						
	25/15	AR-GF	1.00								24.2	114%						
	25/15	AR-GF	2.00								26.8	126%						
	25/15	AR-GF	3.00								23.9	112%						
[29]	-	-	0.00 ^(1)^	-	181	100%					32.3	100%					5.10	100%
	6/14.2	AR-GF	0.50 ^(1)^	511	158	87%					35.4	109%					5.16	101%
	6/14.2	AR-GF	1.00 ^(1)^	511	132	73%					24.5	76%					5.00	98%
	6/14.2	AR-GF	1.50 ^(1)^	511	111	61%					23.7	73%					4.67	92%
	25.4/14.2	AR-GF	0.50 ^(1)^	511	131	72%					33.1	103%					5.26	103%
	25.4/14.2	AR-GF	1.00 ^(1)^	511	99	55%					34.0	105%					5.39	106%
	25.4/14.2	AR-GF	1.50 ^(1)^	511	84	46%					29.9	93%					5.71	112%
[30]	-	-	0.00	-							38.0	100%	2.80	100%			3.50	100%
	6 + 12 + 18/14	AR-GF	0.25	>1700							38.6	102%	3.30	118%			4.95	141%
[30]	6 + 12 + 18/14	AR-GF	0.50	>1700							39.8	105%	3.38	121%			4.93	141%
	6 + 12 + 18/14	AR-GF	0.75	>1700							40.0	105%	3.37	120%			4.82	138%
	6 + 12 + 18/14	AR-GF	1.00	>1700							39.3	104%	3.28	117%			4.78	137%
[32]	-	-	0.00 ^(3)^	-							49.6	100%					6.30	100%
	25/16	AR-GF	1.50 ^(3)^								46.6	94%					10.22	162%
	25/16	AR-GF	2.50 ^(3)^								47.3	95%					10.29	163%
	25/16	AR-GF	4.04 ^(3)^														19.81	314%
	25/16	AR-GF	5.72 ^(3)^														25.04	397%
[33]	-	-	0.00	-							53.4	100%	3.03	100%				
	30/800	SF	0.25	1150							52.6	98%	3.88	128%				
	12/N.I.	E-GF	0.25								54.8	103%	3.61	119%				
[34]	-	-	0.00 ^(1)^	-					13.5	100%	20.8	100%	1.85	100%			2.41	100%
	6 + 18/13	E-GF	0.70 ^(1)^						15.2	113%	22.0	106%	2.20	119%			2.92	121%
[35]	- ^(I)^	-	0.00	-														100%
	13/18 ^(I)^	GF	0.50	2000														126%
	13/18 ^(I)^	GF	1.00	2000														133%
	13/18 ^(I)^	GF	1.50	2000														137%
	13/18 ^(I)^	GF	2.00	2000														139%
	13/18 ^(I)^	GF	2.50	2000														152%
	13/18 ^(I)^	GF	3.00	2000														159%
	- ^(II)^	-	0.00	-														100%
	13/18 ^(II)^	GF	0.50	2000														133%
	13/18 ^(II)^	GF	1.00	2000														141%
	13/18 ^(II)^	GF	1.50	2000														144%
	13/18 ^(II)^	GF	2.00	2000														159%
	13/18 ^(II)^	GF	2.50	2000														162%
	13/18 ^(II)^	GF	3.00	2000														169%
[36]	- ^(VC)^	-	0.00	-	55	100%					47.8	100%	3.60	100%			4.36	100%
	35/750 ^(VC)^	SF	0.30	1100	45	82%					51.2	107%	3.89	108%			4.66	107%
	35/750 ^(VC)^	SF	0.60	1100	35	64%					54.2	114%	4.06	113%			5.07	116%
	35/750 ^(VC)^	SF	0.90	1100	27	49%					57.1	120%	4.27	119%			5.41	124%
	6/13 ^(VC)^	AR-GF	0.02	1700	48	87%					50.2	105%	3.78	105%			4.56	105%
	6/13 ^(VC)^	AR-GF	0.03	1700	45	82%					53.7	112%	3.92	109%			4.66	107%
	6/13 ^(VC)^	AR-GF	0.04	1700	42	76%					57.3	120%	4.10	114%			5.13	118%
	- ^(SCC)^	-	0.00	-	740^(2)^	100%	4.3	100%			54.4	100%	3.85	100%			4.74	100%
	35/750 ^(SCC)^	SF	0.30	1100	690^(2)^	93%	7.2	167%			58.6	108%	4.19	109%			5.25	111%
[36]	35/750 ^(SCC)^	SF	0.60	1100	655 ^(2)^	89%	9.5	221%			62.3	114%	4.45	116%			5.63	119%
	35/750 ^(SCC)^	SF	0.90	1100	590 ^(2)^	80%	11.3	263%			65.4	120%	4.65	121%			5.74	121%
	6/13 ^(SCC)^	AR-GF	0.02	1700	720 ^(2)^	97%	5.3	123%			57.6	106%	4.13	107%			5.05	107%
	6/13 ^(SCC)^	AR-GF	0.03	1700	700 ^(2)^	95%	6.4	149%			61.3	113%	4.24	110%			5.17	109%
	6/13 ^(SCC)^	AR-GF	0.04	1700	680 ^(2)^	92%	7.4	172%			65.6	120%	4.39	114%			5.61	118%
[37]	-	-	0.00	-							45.0	100%	2.90	100%				
	21.7/N.I.	RGF	1.20								38.5	86%	3.75	129%				
	17.2/N.I.	RGF	1.20								41.0	91%	3.00	103%				
	21.2/N.I.	RGF	1.20								40.0	89%	2.75	95%				
	19.5/N.I.	RGF	1.20								39.0	87%	3.25	112%				
	25.4/N.I.	E-GF	1.20								43.5	97%	4.00	138%				
[38]	-	-	0.00	-							25.9	100%					2.15	100%
	12/N.I.	AR-GF	0.19								36.7	142%					3.21	149%
	12/N.I.	AR-GF	0.37								34.5	133%					3.10	144%
	12/N.I.	AR-GF	0.74								39.8	154%					4.12	192%
	40/N.I.	AR-GF	0.19								27.1	105%					4.50	209%
[49]	-	-	0.00	-	22	100%			31.5	100%	47.0	100%	2.86	100%			8.00	100%
	49/838 × 555	GF	0.02	1700	20	91%			31.8	101%	53.0	113%	3.51	123%			9.00	113%
	49/838 × 555	GF	0.04	1700	18	82%			31.9	101%	57.0	121%	4.11	144%			10.00	125%
	49/838 × 555	GF	0.07	1700	15	68%			32.1	102%	61.0	130%	5.65	198%			10.40	130%
[50]	-	-	0.00 ^(1)^	-	110	100%					23.0	100%	2.47	100%			4.00	100%
	12/15	AR-D GF	0.50 ^(1)^		96	87%					18.0	78%	2.19	89%			5.00	125%
	12/15	AR-D GF	1.00 ^(1)^		95	86%					22.0	96%	2.23	90%			5.00	125%
	12/15	AR-D GF	1.50 ^(1)^		92	84%					26.0	113%	2.74	111%			6.00	150%
	12/15	AR-D GF	2.00 ^(1)^		90	82%					22.0	96%	2.57	104%			6.00	150%
	12/15	AR-D GF	2.50 ^(1)^		88	80%					22.0	96%	2.30	93%			5.00	125%
	12/15	AR-D GF	3.00 ^(1)^		85	77%					20.0	87%	2.27	92%			4.00	100%
	12/15	AR-D GF	3.50 ^(1)^		84	76%					18.0	78%	2.32	94%			4.00	100%
[51]	-	-	0.00	-							30.1	100%					9.56	100%
	12/14	GF	0.33								41.3	137%					13.70	143%
	12/14	GF	0.67								32.2	107%					16.80	176%
	12/14	GF	1.00								28.7	95%					18.80	197%
[52]	-	-	0.00 ^(1)^	-							26.9	100%						
	15/12	AR-D GF	1.50 ^(1)^								29.0	108%						
[53]	-	-	0.00	-							47.5	100%	3.19	100%			6.55	100%
	36/450	SF	0.50								53.8	113%	3.58	112%			6.78	104%
[53]	36/450	SF	0.75								54.7	115%	3.65	114%			6.91	105%
	36/450	SF	1.00								56.3	118%	3.79	119%			7.10	108%
	36/450	SF	1.25								53.9	114%	3.55	111%			6.85	105%
	12/200	GF	0.50								52.0	109%	3.35	105%			6.83	104%
	12/200	GF	1.00								53.1	112%	3.43	108%			6.92	106%
	12/200	GF	1.50								54.3	114%	3.51	110%			7.05	108%
	12/200	GF	2.00								53.7	113%	3.40	107%			6.98	107%
[54]	- ^(I)^	-	0.00	-					10.3	100%	21.7	100%					1.10	100%
	13/18 ^(I)^	GF	1.00	2000					10.9	106%	25.6	118%					3.80	345%
	13/18 ^(I)^	GF	1.50	2000					10.8	105%	26.7	123%					4.90	445%
	13/18 ^(I)^	GF	2.00	2000					10.7	104%	26.2	121%					7.60	691%
	19/18 ^(I)^	GF	1.00	2000					10.4	101%	26.9	124%					3.00	273%
	19/18 ^(I)^	GF	1.50	2000					10.8	105%	26.1	120%					5.00	455%
	19/18 ^(I)^	GF	2.00	2000					10.9	106%	25.6	118%					7.30	664%
	- ^(II)^	-	0.00	-					22.3	100%	50.4	100%					2.10	100%
	13/18 ^(II)^	GF	1.00	2000					21.9	98%	55.6	110%					5.40	257%
	13/18 ^(II)^	GF	1.50	2000					19.7	88%	55.2	110%					9.00	429%
	13/18 ^(II)^	GF	2.00	2000					19.4	87%	58.6	116%					10.10	481%
	19/18 ^(II)^	GF	1.00	2000					21.5	96%	53.0	105%					7.60	362%
	19/18 ^(II)^	GF	1.50	2000					19.9	89%	50.4	100%					9.70	462%
	19/18 ^(II)^	GF	2.00	2000					19.2	86%	55.7	111%					10.80	514%

*E_cm_*—concrete modulus of elasticity; *f_c_*—compressive strength; *f_spl_*—splitting tensile strength; *f_t_*—uniaxial tensile strength; *f_fl_*—flexural tensile strength; AR-GF—alkali-resistant glass fibers; AR-D-GF—alkali resistant-water dispersed glass fibers; E-GF—insulation to electricity glass fibers; (R)GF—(recycled) glass fibers; PPF—polypropylene fibers; SF—steel fibers; ^(1)^—% of cement weight; ^(2)^—slump flow (spread); ^(3)^—% of concrete weight; ^(I)^—mixture type I; ^(II)^—mixture type II; ^(III)^—mixture type III; ^(IV)^—mixture type IV; ^(VC)^—vibrated concrete; ^(SCC)^—self-compacting concrete.

**Table 5:** Percentage of mass loss of rebar due to corrosion for PC and GFRC [27].

***V_f_* [%]**	0.0	0.5	1.5	2.0	2.5	3.0
**Mass loss [%]**	15.6	13.5	12.4	9.24	11.54	11.83

**Table 6:** Effect of GF addition on different concrete properties.

Property	Effect
Workability	−
Crack limitation, shrinkage	++
Modulus of elasticity	±
Compressive strength	±
Tensile strength	+
Post-cracking behavior, ductility, toughness	++
Impact resistance	++
Spalling and fire resistance	++
Abrasion resistance	+
Thermal conductivity	+
Durability	++
Permeability	+
Freeze-thaw resistance	++
Corrosion resistance	++
Bonding strength	+
Eco-friendly, economic	+

− negative; ± difficult to assess; + positive; ++ very positive.

## Data Availability

Not applicable.
